# Efficient Synthesis of Hydrolytically Degradable Block Copolymer Nanoparticles via Reverse Sequence Polymerization‐Induced Self‐Assembly in Aqueous Media

**DOI:** 10.1002/anie.202309526

**Published:** 2023-08-10

**Authors:** Matthew A. H. Farmer, Osama M. Musa, Steven P. Armes

**Affiliations:** ^1^ Department of Chemistry The University of Sheffield Brook Hill S3 7HF Sheffield South Yorkshire UK; ^2^ Ashland Specialty Ingredients 1005 US 202/206 08807 Bridgewater NJ USA

**Keywords:** Aqueous Dispersions, Block Copolymer Nanoparticles, Hydrolytic Degradation, RAFT Polymerization, Reverse Sequence Polymerization-Induced Self-Assembly

## Abstract

Hydrolytically degradable block copolymer nanoparticles are prepared via *reverse sequence* polymerization‐induced self‐assembly (PISA) in aqueous media. This efficient protocol involves the reversible addition‐fragmentation chain transfer (RAFT) polymerization of *N,N′*‐dimethylacrylamide (DMAC) using a monofunctional or bifunctional trithiocarbonate‐capped poly(ϵ‐caprolactone) (PCL) precursor. DMAC monomer is employed as a co‐solvent to solubilize the hydrophobic PCL chains. At an intermediate DMAC conversion of 20–60 %, the reaction mixture is diluted with water to 10–25 % w/w solids. The growing amphiphilic block copolymer chains undergo nucleation to form sterically‐stabilized PCL‐core nanoparticles with PDMAC coronas. ^1^H NMR studies confirm more than 99 % DMAC conversion while gel permeation chromatography (GPC) studies indicate well‐controlled RAFT polymerizations (*M*_w_/*M*_n_≤1.30). Transmission electron microscopy (TEM) and dynamic light scattering (DLS) indicate spheres of 20–120 nm diameter. As expected, hydrolytic degradation occurs within days at 37 °C in either acidic or alkaline solution. Degradation is also observed in phosphate‐buffered saline (PBS) (pH 7.4) at 37 °C. However, no degradation is detected over a three‐month period when these nanoparticles are stored at 20 °C in deionized water (pH 6.7). Finally, PDMAC_30_‐PCL_16_‐PDMAC_30_ nanoparticles are briefly evaluated as a dispersant for an agrochemical formulation based on a broad‐spectrum fungicide (azoxystrobin).

## Graphical abstract

Hydrolytically degradable block copolymer nanoparticles are prepared via *reverse sequence* polymerization‐induced self‐assembly (PISA) in aqueous media. This one‐pot protocol involves reversible addition‐fragmentation chain transfer (RAFT) polymerization of *N,N′*‐dimethylacrylamide (DMAC) using a monofunctional or bifunctional trithiocarbonate‐capped poly(ϵ‐caprolactone) (PCL) precursor; DMAC monomer initially acts as a co‐solvent to solubilize the hydrophobic PCL chains.

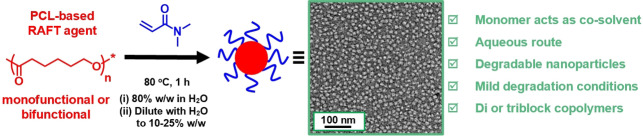

## Introduction

Over the past decade or so, polymerization‐induced self‐assembly (PISA) has become an established and widely used technique for the rational synthesis of block copolymer nano‐objects.[^1^, ^2^, ^3^, ^4^, ^5^, ^6^, ^7^] Conventional PISA involves growing an insoluble block from a soluble precursor block in a suitable solvent. At some critical degree of polymerization (DP), micellar nucleation occurs to form monomer‐swollen nascent nanoparticles, which then act as the locus for the remaining polymerization to produce sterically‐stabilized nanoparticles.[^8^, ^9^] Traditionally, PISA has been conducted using vinyl monomers, which inevitably leads to non‐degradable nanoparticles. This is an unfortunate limitation, because such nanoparticles offer a wide range of potential applications, including dispersants for agrochemicals,[^10^, ^11^] emulsifiers for the production of Pickering nanoemulsions,[^12^, ^13^, ^14^] thermoresponsive biocompatible hydrogels for either cell culture or long‐term stem cell storage,[^15^, ^16^] and low‐viscosity lubricants for automotive engine oils.^[17]^ In each case, nanoparticle degradability would be a desirable “value‐added” feature.

Given the strong global demand for more environmentally‐friendly polymers, several attempts have been made to develop PISA routes to degradable nanoparticles. For example, Nicolas and co‐workers introduced hydrolytically cleavable bonds into copolymer chains by using reversible addition‐fragmentation chain transfer (RAFT) polymerization^[18]^ to statistically copolymerize cyclic ketal acetals (CKA) such as 2‐methylene‐1,3‐dioxepane (MDO) with either styrene or (meth)acrylic monomers.[^19^, ^20^, ^21^] Similarly, Roth and co‐workers[^22^, ^23^, ^24^] (and more recently other research groups[^25^, ^26^, ^27^, ^28^, ^29^]) reported the statistical copolymerization of dibenzo[c,e]oxepane‐5‐thione (DOT) with various vinyl monomers. Only a relatively low level of incorporation of such a cyclic comonomer is required to obtain oligomers after hydrolytic degradation. However, this approach suffers from some technical problems. First, such cyclic comonomers invariably require multi‐step syntheses for which the overall yield is relatively low. Moreover, the radical ring‐opening copolymerization of CKAs with vinyl monomers often retards the overall rate of polymerization, which makes full comonomer conversion difficult to achieve. Finally, such cyclic comonomers are typically water‐insoluble, which may complicate their use in aqueous formulations. However, significant progress has been recently made with regard to some of these problems.[^21^, ^26^, ^30^] In particular, RAFT aqueous emulsion copolymerization of DOT with styrene has been achieved with full comonomer conversion and a relatively narrow molecular weight distribution.^[30]^

In view of the above problems, alternative approaches to degradable nanoparticles have been developed. Notably, Lecommandoux's group reported the synthesis of degradable polypeptide‐based diblock copolymers via ring‐opening polymerization of *N*‐carboxyanhydrides (NCA) using a poly(ethylene glycol)‐based (PEG) precursor.[^31^, ^32^] Remarkably, such syntheses can be conducted directly in aqueous media. However, the synthesis of NCA monomers usually requires the use of highly toxic phosgene.^[33]^ Nevertheless, this approach enabled the synthesis of degradable rod‐like nanoparticles.[^31^, ^32^] Similarly, Du and co‐workers reported the preparation of well‐defined diblock copolymer vesicles via NCA polymerization when using a monofunctional PEG precursor, albeit in THF rather than water^[34]^

**Scheme 1:**
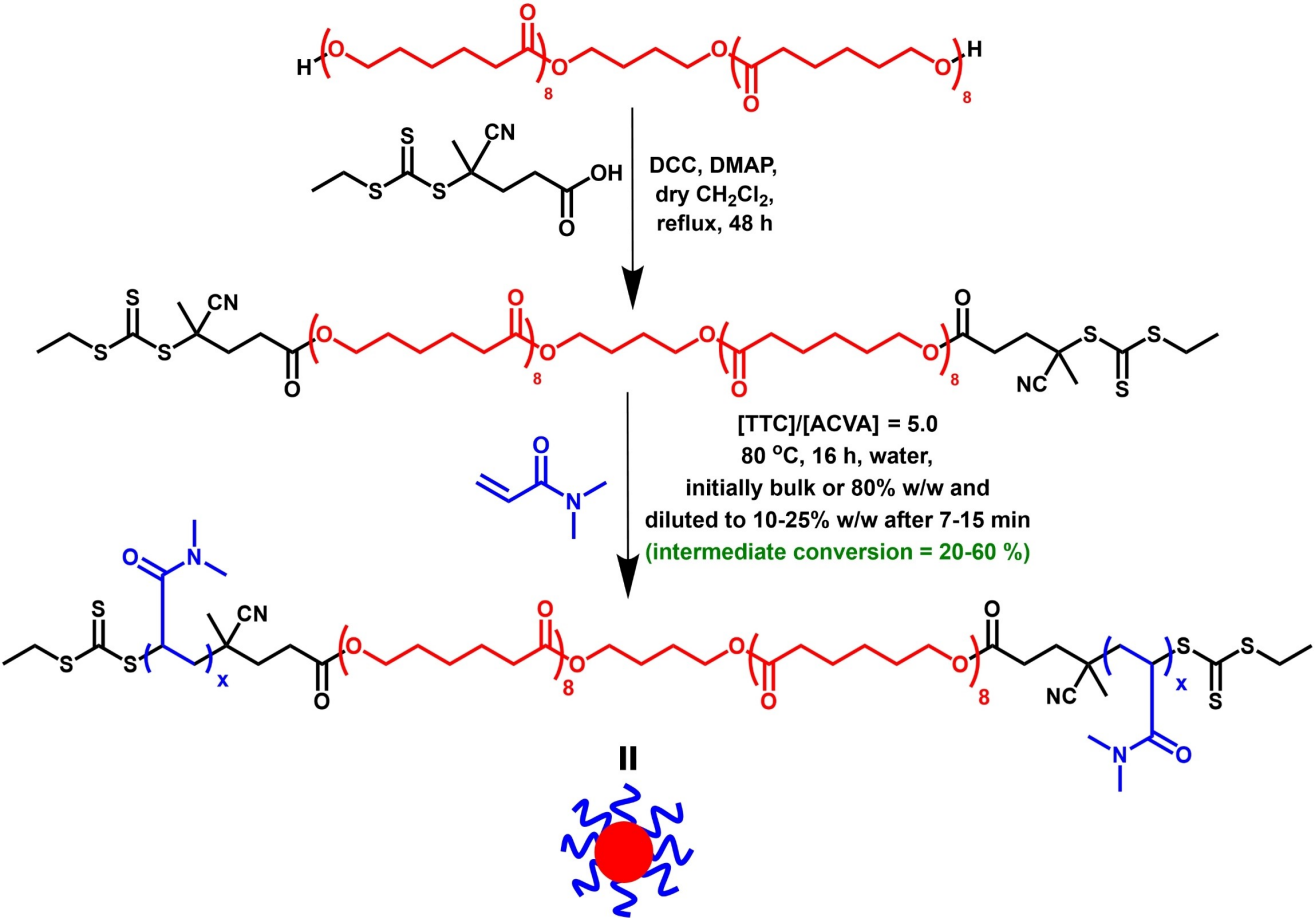
Synthesis of a bifunctional trithiocarbonate‐capped RAFT agent (TTC‐PCL_16_‐TTC) via DCC/DMAP‐catalyzed esterification of a dihydroxy‐capped PCL precursor using a carboxylic acid‐functionalized RAFT agent (CEPA). Subsequently, PDMAC_x_‐PCL_16_‐PDMAC_x_ nanoparticles are prepared at 80 °C via *reverse sequence* PISA. Initially, the RAFT polymerization of DMAC is conducted either in the bulk or at 80 % w/w solids, with subsequent dilution to 10–25 % w/w solids using deoxygenated deionized water at a suitable intermediate DMAC conversion. Conditions: [TTC]/[ACVA] molar ratio=5.

Recently, we reported a novel approach to PISA known as *reverse sequence* PISA.[^35^, ^36^] This involved the RAFT aqueous dispersion polymerization of 2‐hydroxypropyl methacrylate (HPMA) using an anionic water‐soluble RAFT agent to produce charge‐stabilized PHPMA latexes. Subsequent chain extension of these relatively large precursor particles using a water‐miscible methacrylic monomer leads to the formation of much smaller sterically‐stabilized spherical nanoparticles. Such aqueous formulations are rather counter‐intuitive because they involve synthesis of the hydrophobic block first. Herein we report a new type of *reverse sequence* PISA formulation that provides convenient access to hydrolytically degradable nanoparticles via an aqueous protocol. This involves initial solubilization of a hydrophobic trithiocarbonate‐capped poly(ϵ‐caprolactone) (PCL) precursor with the aid of a suitable water‐miscible monomer (*N,N′*‐dimethylacrylamide, DMAC), see Scheme 1. The DMAC initially serves as a co‐solvent to ensure dissolution of the otherwise water‐insoluble PCL. Subsequently, RAFT polymerization of DMAC is conducted at 80 °C either in concentrated aqueous solution or in the bulk. Once a sufficiently long PDMAC block has been grown, the homogeneous reaction mixture is diluted with water to 10–25 % w/w solids. As DMAC monomer is consumed, the growing amphiphilic block copolymer chains undergo nucleation to form sterically‐stabilized PCL‐core spherical nanoparticles. These nanoparticles are characterized in terms of their morphology and size by transmission electron microscopy (TEM) and dynamic light scattering (DLS) respectively. Moreover, their long‐term hydrolytic degradation in aqueous solution is studied under various conditions. Finally, selected nanoparticles were evaluated as a putative dispersant for the formulation of a well‐known agrochemical compound (azoxystrobin, a broad‐spectrum fungicide).

## Results and Discussion

Recently, various research groups have reported the statistical copolymerization of cyclic monomers with vinyl monomers to produce hydrolytically degradable copolymer backbones.[^19^, ^20^, ^21^, ^22^, ^26^, ^29^, ^30^, ^31^, ^34^, ^37^, ^38^] In principle, this approach could form part of the solution to the global challenge of plastic waste.[^39^, ^40^] Accordingly, it has been evaluated for solution polymerization,[^22^, ^37^] conventional aqueous emulsion polymerization[^29^, ^41^, ^42^] and for RAFT‐mediated PISA.[^19^, ^20^, ^30^] Nevertheless, this strategy currently suffers from several potential disadvantages, as discussed above. Herein, we propose an alternative route to hydrolytically degradable block copolymer nanoparticles based on a new *reverse sequence* PISA formulation, as summarized in Scheme 1.

Both monohydroxy‐capped (Scheme S1) and dihydroxy‐capped PCL precursors were derivatized using a carboxylic acid‐functionalized RAFT agent (CEPA) via esterification catalyzed by *N,N′*‐dicyclohexylcarbodiimide (DCC) and 4‐(dimethylamino)pyridine (DMAP). CEPA was preferred to other well‐known RAFT agents because it confers an ethyl end‐group, as opposed to more hydrophobic n‐dodecyl or aromatic end‐groups.[^43^, ^44^, ^45^, ^46^] In principle, this should aid solubilization of the hydrophobic PCL precursor when using DMAC monomer as a co‐solvent. Indeed, the TTC‐PCL_16_‐TTC precursor (where TTC denotes the trithiocarbonate end‐groups) is fully soluble in this monomer at 20 °C when 40 or more molar equivalents of DMAC are added (see Figure S1). However, for syntheses conducted using an 80 % w/w aqueous solution of DMAC (see Scheme 1), the TTC‐PCL_16_‐TTC precursor is insoluble at 20 °C and only becomes fully dissolved on heating up to the polymerization temperature of 80 °C, see Figure S2. Similar solubility behavior was observed for the PCL_42_‐TTC precursor (see Figures S3 and S4).

**Figure 1:**
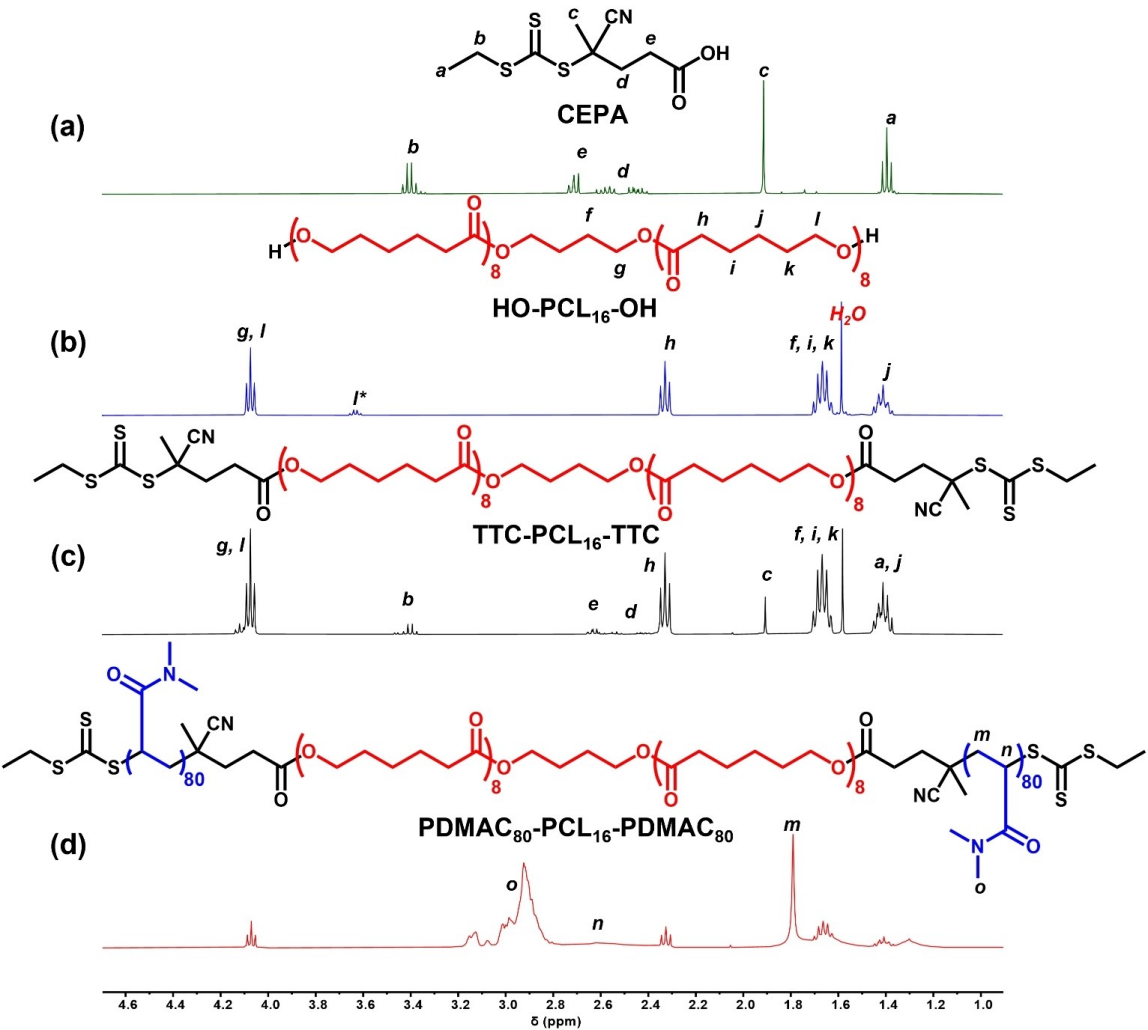
^1^H NMR spectra (CD_2_Cl_2_) recorded for (a) the CEPA RAFT agent, (b) the dihydroxy‐capped PCL_16_ precursor (where l* represents the terminal l protons), (c) the bifunctional TTC‐PCL_16_‐TTC RAFT agent and (d) a PDMAC_80_‐PCL_16_‐PDMAC_80_ triblock copolymer.

^1^H NMR spectroscopy was used to determine a mean degree of esterification of 98±1 % for this bifunctional precursor by comparing the integrated proton signal at 1.91 ppm assigned to the methyl group of the RAFT agent with the unique PCL backbone signals at 4.07, 2.33 and 1.66 ppm (see Figure 1). This technique was also used to determine the mean degree of polymerization of each of the three monohydroxy‐capped PCL precursors and to confirm the mean degree of polymerization of the as‐received dihydroxy‐capped PCL precursor (see Figures S5–S8). Finally, high degrees of esterification were confirmed for the corresponding three monofunctional PCL_21_‐TTC (98±3 %), PCL_29_‐TTC (96±2 %) and PCL_42_‐TTC (100±1 %) precursors, see Figures S9–S12. Furthermore, gel permeation chromatography (GPC) analysis using a UV detector indicated that no residual CEPA RAFT agent remained after purification (see Figure S13).

Initial DMAC polymerizations were conducted in the bulk to ensure complete dissolution of the relevant hydrophobic PCL precursor. Subsequently, it was discovered that homogeneous reaction solutions could also be obtained in the presence of a small amount of water (see Scheme 1). Once sufficient DMAC had been polymerized, a significant increase in the solution viscosity was observed. This visual cue was used to indicate when to add the deoxygenated deionized water to produce a more dilute reaction mixture. This dilution step produced an aqueous dispersion of PCL‐core nanoparticles. Currently, it is not known whether any nascent nanoparticles are formed prior to dilution. Analytical techniques such as DLS or TEM require substantial dilution of the reaction mixture: this would inevitably induce nanoparticle formation (if it had not already occurred) because the solvency for the PCL block is reduced. In principle, time‐resolved small‐angle X‐ray scattering (SAXS) could determine whether nucleation had occurred prior to dilution of the reaction mixture. However, such experiments require access to a synchrotron X‐ray source and are beyond the scope of the present study.

**Figure 2:**
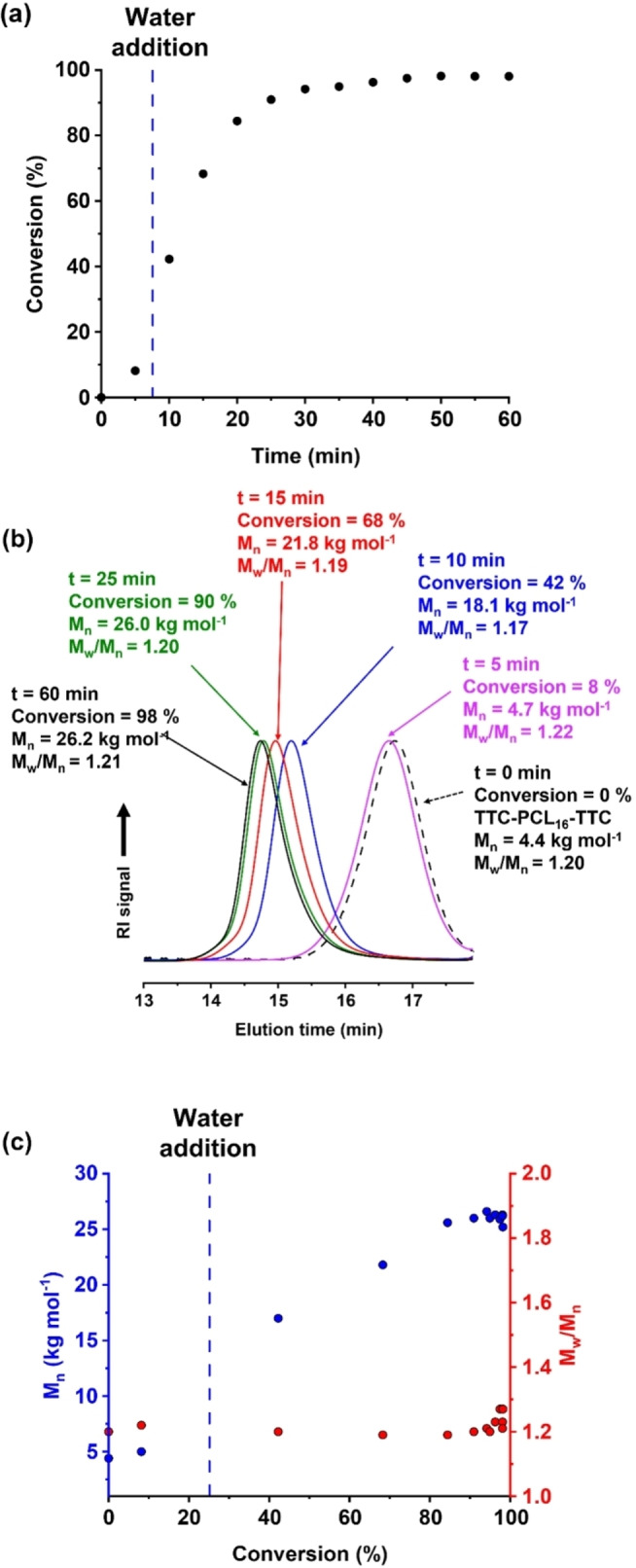
(a) Conversion vs. time curve (black points) obtained by ^1^H NMR analysis for the *reverse sequence* PISA synthesis of PDMAC_80_‐PCL_16_‐PDMAC_80_ nanoparticles at 80 °C. Initially, the RAFT polymerization of DMAC was conducted in the bulk with subsequent dilution to 10 % w/w solids using deoxygenated deionized water after 7.5 min (or ≈25 % DMAC conversion). Conditions: [TTC]/[ACVA] molar ratio=5.0. (b) Selected DMF GPC curves (refractive index detector) and (c) the corresponding *M*_n_ (blue points) and *M*_w_/*M*_n_ (red points) data determined during this *reverse sequence* PISA synthesis.

A kinetic study was conducted when targeting a PDMAC DP of 80 using a TTC‐PCL_16_‐TTC precursor at 80 °C. After 7.5 min, the initial bulk polymerization was diluted with deoxygenated water to produce a 10 % w/w aqueous dispersion of nascent sterically‐stabilized triblock copolymer nanoparticles (Figure 2a). The reaction mixture was periodically sampled and the resulting aliquots were analyzed by ^1^H NMR spectroscopy. On addition of water after 7.5 min, the instantaneous DMAC conversion was estimated to be 25 % and a final monomer conversion of 98 % was achieved after 50 min at 80 °C. Interestingly, dilution of the reaction mixture from the bulk to 10 % w/w solids did not result in any discernible reduction in the rate of polymerization. This is presumably because the polymerization of acrylamides proceeds much faster in dilute aqueous solution than in the bulk.[^47^, ^48^] For example, Büback and co‐workers reported a nine‐fold increase in the propagation rate constant (*k_p_*) for the free radical polymerization of DMAC in 20 % aqueous solution at 80 °C compared to the corresponding bulk polymerization at the same temperature.^[48]^

Unlike conventional aqueous PISA formulations, no dramatic increase in the rate of polymerization is observed after micellar nucleation.^[9]^ This is simply because the growing PDMAC steric stabilizer chains are located on the outside of the nanoparticles, so there is no tangible benefit if the nanoparticle cores become swollen with unreacted DMAC monomer. GPC analysis of this *reverse sequence* PISA formulation indicated a linear evolution in molecular weight for the triblock copolymer chains with conversion. Moreover, a very high blocking efficiency was observed and the molecular weight distribution remained relatively narrow (*M*_w_/*M*_n_<1.30), indicating a well‐controlled RAFT polymerization (see Figure 2b and 2c).

**Figure 3:**
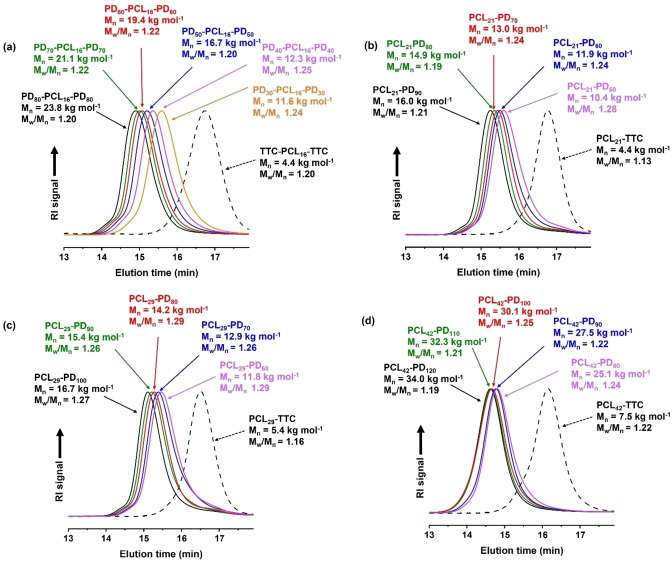
DMF GPC curves (refractive index detector) recorded for a series of block copolymers prepared by *reverse sequence* PISA in aqueous media using an ACVA initiator at 80 °C. (a) Bifunctional TTC‐PCL_16_‐TTC precursor and a corresponding series of PDMAC_x_‐PCL_16_‐PDMAC_x_ triblock copolymers. (b) Monofunctional PCL_21_‐TTC precursor and a corresponding series of PCL_21_‐PDMAC_z_ diblock copolymers. (c) Monofunctional PCL_29_‐TTC precursor and a corresponding series of PCL_29_‐PDMAC_z_ diblock copolymers. (d) Monofunctional PCL_42_‐TTC precursor and a corresponding series of PCL_42_‐PDMAC_z_ diblock copolymers.

Having confirmed the efficient synthesis of well‐defined block copolymers by this new *reverse sequence* PISA route, a library of PDMAC_x_‐PCL_16_‐PDMAC_x_ triblock copolymers and PCL_y_‐PDMAC_z_ diblock copolymers was prepared by systematically varying the target DP of each block (see Table S1). Representative GPC traces are shown in Figure 3a when using a bifunctional TTC‐PCL_16_‐TTC precursor to target a range of PDMAC DPs for initial bulk polymerizations conducted at 80 °C; each reaction mixture was then diluted to 10 % w/w solids using deoxygenated deionized water within 10 min. In all cases, aqueous colloidal dispersions of block copolymer nanoparticles were obtained. High blocking efficiencies were observed when using either a bifunctional TTC‐PCL_16_‐TTC precursor or a monofunctional PCL_21_‐TTC precursor, as indicated by UV GPC analysis at λ=305 nm (see Figure S14). However, UV GPC analysis revealed tailing towards low molecular weight when using either PCL_29_‐TTC or PCL_42_‐TTC, which suggests a small fraction of dead chains.

Furthermore, at least 99% DMAC conversion was obtained after 16 h as judged by ^1^H NMR spectroscopy. Similarly, a series of monofunctional PCL_y_‐TTC precursors were employed to target a range of PDMAC DPs using the same synthetic protocol (see Figure 3b–d). Again, essentially full DMAC conversion was achieved in each case within 16 h.

**Figure 4:**
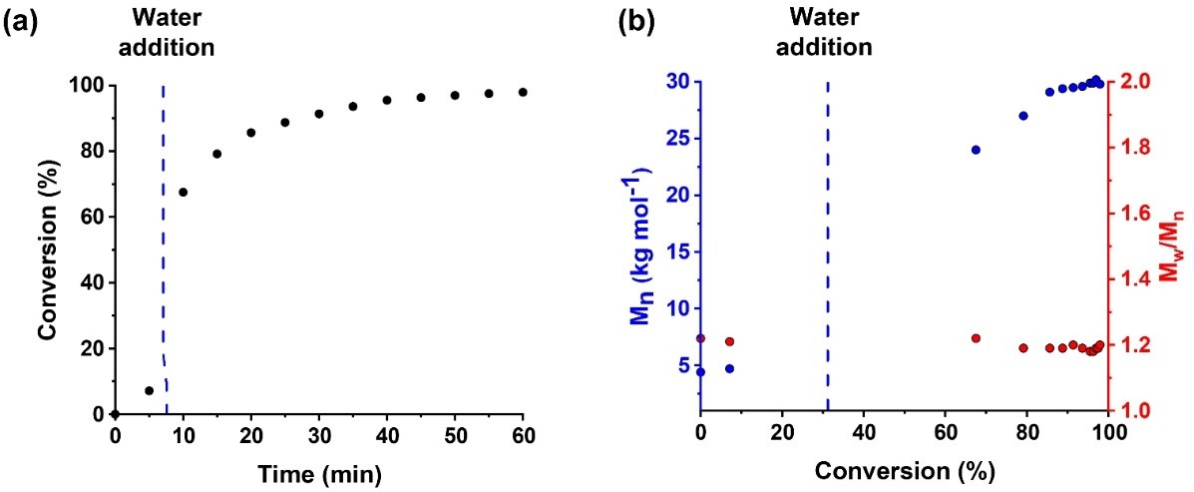
(a) Conversion vs. time curve (black points) obtained by ^1^H NMR spectroscopy for the *reverse sequence* PISA synthesis of PDMAC_80_‐PCL_16_‐PDMAC_80_ nanoparticles prepared at 80 °C. Initially, the RAFT polymerization of DMAC was conducted at 80 % w/w solids with subsequent dilution to 10 % w/w solids using deoxygenated deionized water after 7 min (which corresponds to ≈31 % DMAC conversion). Conditions: [TTC]/[ACVA] molar ratio=5.0. (b) The corresponding *M*_n_ (blue points) and *M*_w_/*M*_n_ (red points) data determined via DMF GPC analysis (refractive index detector).

Furthermore, such syntheses could be also conducted at 80 % w/w solids, where a range of PDMAC DPs were targeted using a bifunctional TTC‐PCL_16_‐TTC or a PCL_y_‐TTC precursor (see Table S2). The resulting GPC data were consistent with that produced from polymerizations initiated in the bulk (see Figure S15 and S16). Kinetic analysis of DMAC polymerizations performed in 80 % w/w aqueous solution (followed by dilution to 10 % w/w after 7 min) indicated that good control could also be achieved when a small amount of water was present at the beginning of the polymerization (see Figure 4).

**Figure 5:**
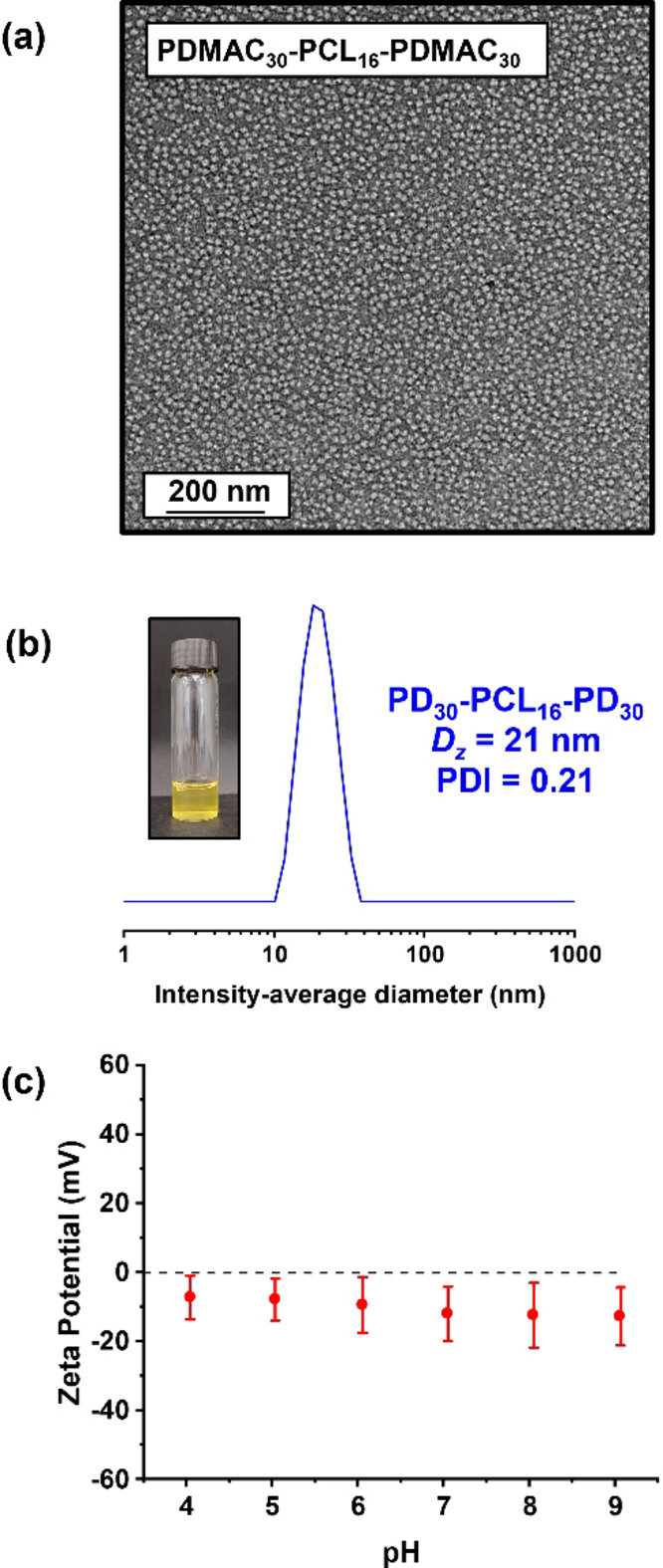
(a) Representative TEM image recorded for an aqueous dispersion of PDMAC_30_‐PCL_16_‐PDMAC_30_ nanoparticles (see entry 6 in Table S2). (b) DLS particle size distribution recorded for the same PDMAC_30_‐PCL_16_‐PDMAC_30_ nanoparticles (inset shows the physical appearance of this aqueous dispersion at 10 % w/w solids). (c) Zeta potential vs. pH curve for a 0.1 % w/w aqueous dispersion of PDMAC_30_‐PCL_16_‐PDMAC_30_ nanoparticles.

TEM analysis of the resulting PDMAC_30_‐PCL_16_‐PDMAC_30_ nanoparticles confirmed a spherical morphology (see Figure 5a) with an estimated number‐average diameter of 16±3 nm (based on digital image analysis of at least 100 nanoparticles), while DLS studies indicated a hydrodynamic z‐average diameter of approximately 21 nm (Figure 5b).

**Figure 6:**
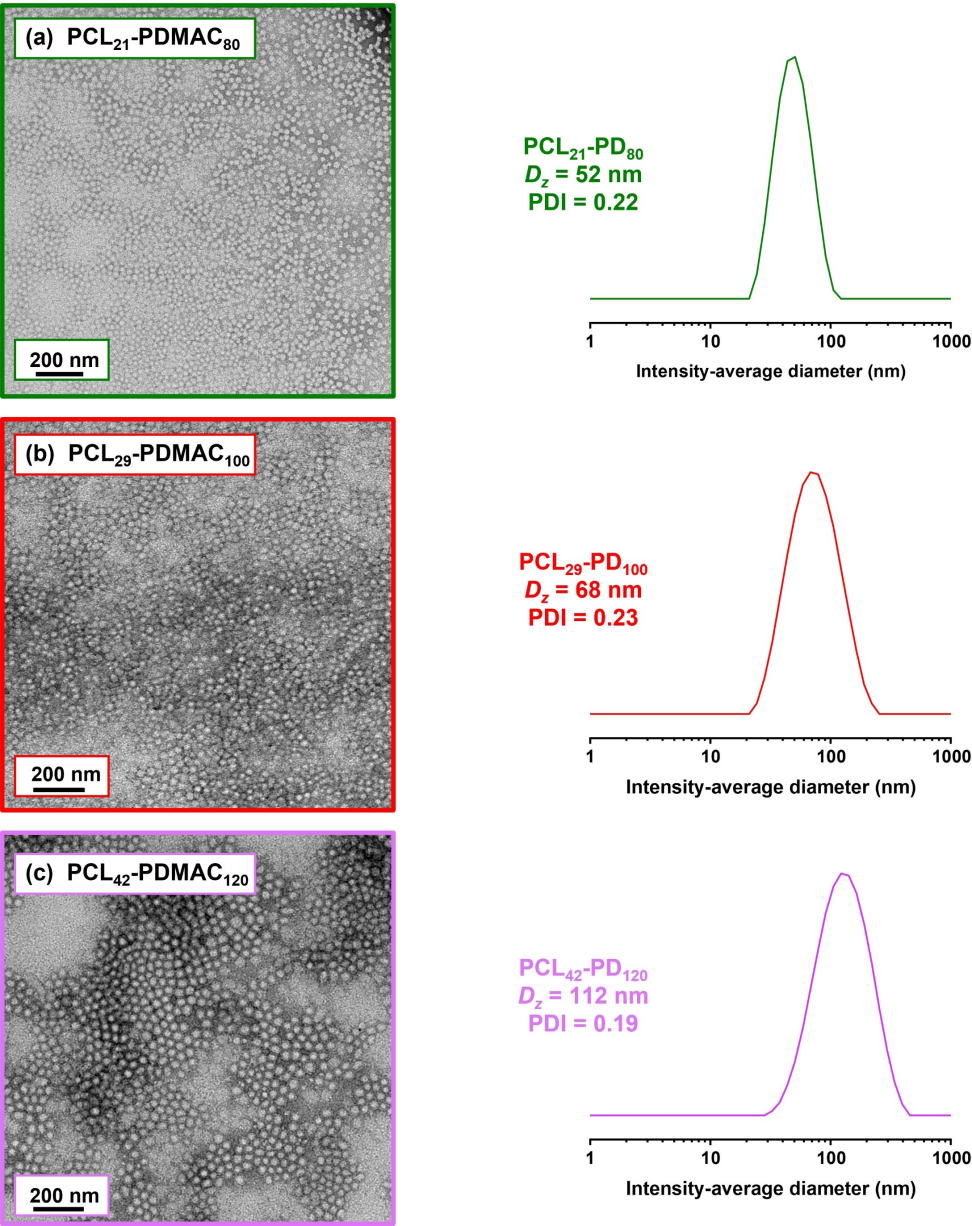
Representative TEM images recorded after drying dilute aqueous dispersions of (a) PCL_21_‐PDMAC_80,_ (b) PCL_29_‐PDMAC_100_ and (c) PCL_42_‐PDMAC_120_ nanoparticles (see entries 9, 14 and 20 in Table S1). Representative DLS particle size distributions recorded for the same three aqueous dispersions.

Aqueous electrophoresis studies revealed zeta potentials close to zero over a wide pH range, as expected given the non‐ionic nature of the PDMAC steric stabilizer chains (Figure 5c). TEM analysis was also performed on the PCL_y_‐PDMAC_z_ diblock copolymer nanoparticles. Spherical nanoparticles were observed in all three cases, with estimated number‐average diameters of 33±5 nm, 40±5 nm and 48±8 nm (based on digital image analysis of at least 100 nanoparticles in each case), see Figure 6. DLS studies indicated a z‐average diameter of approximately 52 nm for the PCL_21_‐PDMAC_80_ nanoparticles and 68 nm for the PCL_29_‐PDMAC_100_ nanoparticles (see Figures 6a and 6b). In contrast, colloidal aggregates of around 112 nm diameter were observed for PCL_42_‐PDMAC_120_, indicating weak flocculation of the primary nanoparticles in this case (see Figure 6c). DLS studies performed on several series of PDMAC_x_‐PCL_16_‐PDMAC_x_ and PCL_y_‐PDMAC_z_ copolymers produced consistent data when varying the target PDMAC DP (see Figures S17–S22). After demonstrating the aqueous synthesis of PCL‐core nanoparticles at 10 % w/w solids, higher final nanoparticle concentrations were investigated. For example, a bifunctional TTC‐PCL_16_‐TTC precursor was used to target a PDMAC DP of 80. The initial bulk polymerization was diluted to 15–25 % w/w solids using deoxygenated deionized water (see Table S3). GPC data indicated that varying the final nanoparticle concentration had no discernible effect on the nature of the copolymer chains (see Figure S23). However, increasing the nanoparticle concentration had a significant effect on the physical appearance of the final colloidal dispersion (see Figure S24). Thus, a free‐flowing dispersion was obtained at 10 % w/w, a highly viscous fluid at 15 % w/w, and a free‐standing gel was obtained at either 20 % w/w or 25 % w/w. The gels were analyzed via shear‐induced polarized light imaging (SIPLI) to determine if gelation was due to the presence of worm‐like nanoparticles.^[49]^ However, no characteristic Maltese cross (indicating the presence of anisotropic particles) was observed for any of these gels, suggesting that gelation is instead due to close‐packed spherical micelles.^[50]^ This was confirmed by diluting the 20 % w/w and 25 % w/w gels to 10 % w/w. As expected, degelation occurred on dilution below the minimum copolymer concentration required for spherical micelle gels. Moreover, DLS studies indicate a monotonic increase in the z‐average diameter and a gradual broadening of the particle size distribution on raising the nanoparticle concentration from 10 % w/w to 25 % w/w solids.

### Hydrolytic Degradation of Block Copolymer Nanoparticles

**Figure 7:**
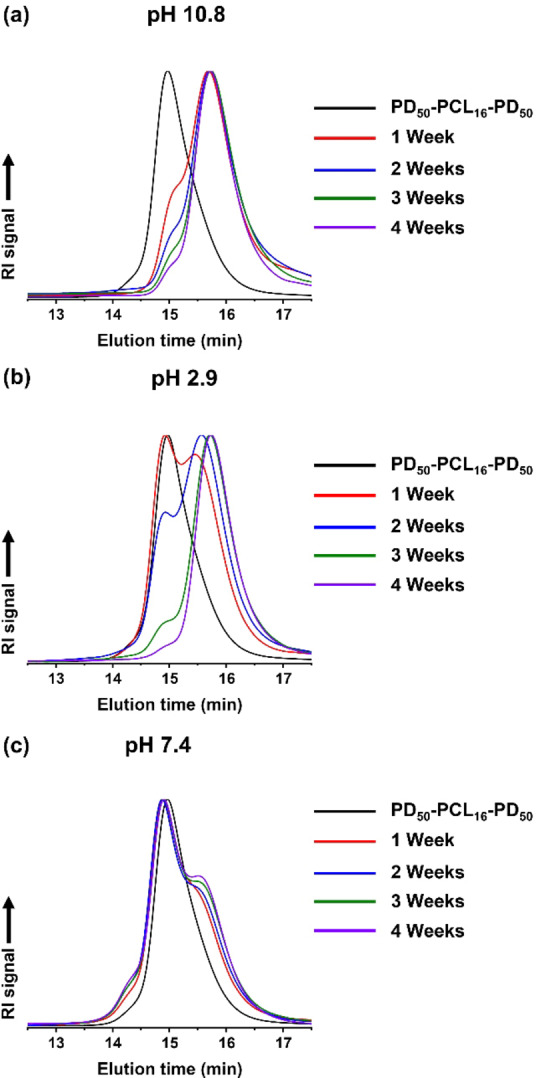
DMF GPC curves (refractive index detector) recorded during the hydrolytic degradation of a 1.0 % w/w aqueous dispersion of PDMAC_50_‐PCL_16_‐PDMAC_50_ nanoparticles at 37 °C for 1–4 weeks at (a) pH 10.8, (b) pH 2.9 and (c) pH 7.4 (PBS buffer).

**Figure 8:**
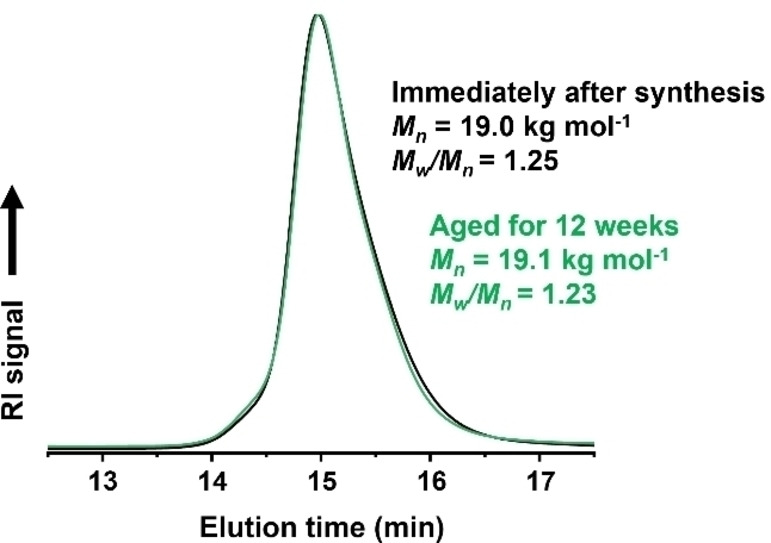
DMF GPC curves (refractive index detector) recorded of a 10 % w/w aqueous dispersion of PDMAC_50_‐PCL_16_‐PDMAC_50_ nanoparticles immediately after synthesis (black curve) and after 12 weeks storage at 20 °C as a 10 % w/w aqueous dispersion at pH 6.7 (green curve).

Selected PCL‐based nanoparticles were subjected to hydrolytic degradation by exposure to an aqueous phosphate‐buffered saline (PBS) buffer (pH 7.4) at 37 °C.[^51^, ^52^, ^53^] For comparison, degradation studies were also conducted at either pH 10.8 or pH 2.9 at the same temperature. For the PDMAC_50_‐PCL_16_‐PDMAC_50_ nanoparticles, hydrolytic degradation was always observed at 37 °C regardless of the solution pH. Random scission of ester bonds within the PCL chains led to an approximate halving of the molecular weight. Subsequently, full degradation of the PCL block produced low molecular weight water‐soluble PDMAC chains. As expected, the fastest rate of ester hydrolysis was observed in alkaline solution (Figure 7). It is perhaps worth emphasizing that the hydrolysis conditions employed herein are much milder than those typically reported in the literature for hydrolytically degradable copolymers prepared via statistical copolymerization of cyclic monomers (e.g. CKAs or DOT) with vinyl monomers.[^19^, ^22^, ^28^] For example, Nicolas and co‐workers employed 0.45 M KOH (pH 14) at 37 °C for up to 28 days for the forced hydrolysis of poly[oligo(ethylene glycol) methyl ether methacrylate]‐b‐poly[(lauryl methacrylate)‐co‐(2‐methylene‐4‐ phenyl‐1,3‐dioxolane)] (POEGMA‐b‐P(LMA‐co‐MPDL)) nanoparticles.^[21]^ A 10 % w/w aqueous dispersion of the same batch of PDMAC_50_‐PCL_16_‐PDMAC_50_ nanoparticles remained colloidally stable after aging for 12 weeks at 20 °C (Figure 8). Indeed, a slightly narrower intensity‐average particle size distribution was observed (see Figure S25).

Finally, degradation studies were also performed on PCL_21_‐PDMAC_70_ and PCL_42_‐PDMAC_120_ diblock copolymer nanoparticles in basic, acidic or neutral (PBS; pH 7.4) solution (see Figures S26 and S27). Like the analogous triblock copolymer nanoparticles, hydrolytic degradation occurred in all cases. Perhaps surprisingly, complete degradation of the PCL cores was observed after four weeks in PBS solution at 37 °C, whereas only partial degradation was observed for the triblock copolymer nanoparticles within the same time scale. As with the triblock copolymer, both types of diblock copolymer chains remained intact as judged by GPC analysis when stored as nanoparticle dispersions in deionized water at 20 °C (see Figure S26 and S27).

### Use of PDMAC_30_‐PCL_16_‐PDMAC_30_ Nanoparticles in an Agrochemical Formulation

The hydrolytically degradable nature of these new PCL‐based nanoparticles is highly desirable for various commercial applications. For example, we recently reported that block copolymer nanoparticles prepared using a conventional aqueous PISA formulation can be used as a dispersant to prepare a concentrated aqueous suspension of a broad spectrum fungicide (azoxystrobin) that is widely used within the agrochemical sector. More specifically, wet ball‐milling of ≈76 μm azoxystrobin crystals in the presence of well‐defined poly(glycerol monomethacrylate)‐poly(methyl methacrylate) diblock copolymer nanoparticles of 29 nm diameter led to the formation of a 20 % w/w aqueous suspension of azoxystrobin microparticles of approximately 2 μm diameter.^[11]^ Electron microscopy studies confirmed that the surface of these microparticles was uniformly coated with the nanoparticles, which conferred long‐term stability.^[11]^ However, such nanoparticles possess an all‐methacrylic backbone so they are classified as a non‐degradable nanoplastic under new environmental legislation. Unfortunately, this precludes their use for this agrochemical application and is also problematic for other potential applications in personal care and cosmetics formulations. Nevertheless, in a follow‐up study we established the fundamental design rules for using nanoparticle dispersants in the context of various agrochemical compounds (including five fungicides and a pesticide).[^10^, ^11^] Bearing the latter results in mind, we decided to evaluate the new hydrolytically degradable PDMAC_30_‐PCL_16_‐PDMAC_30_ nanoparticles as a dispersant for azoxystrobin.

**Figure 9:**
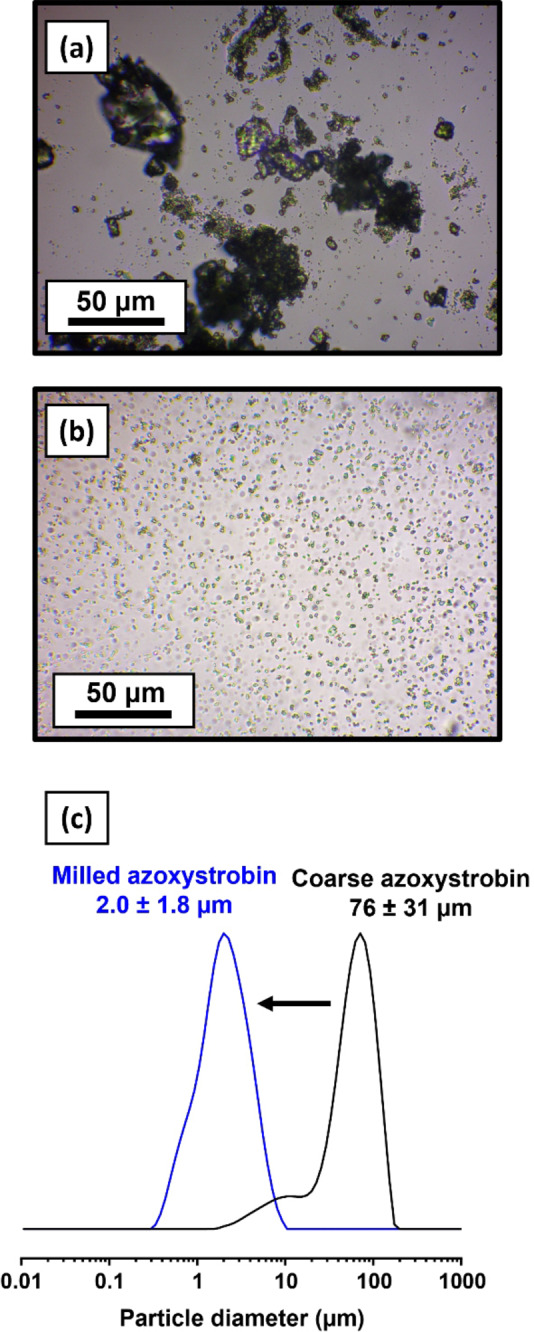
Optical microscopy images recorded for azoxystrobin crystals (a) before and (b) after wet ball‐milling in the presence of PDMAC_30_‐PCL_16_‐PDMAC_30_ nanoparticles (*D*_z_=21 nm diameter). (c) Laser diffraction particle size distributions recorded for the initial coarse azoxystrobin crystals and the much finer nanoparticle‐coated azoxystrobin microparticles obtained after wet ball‐milling for 30 min at 20 °C.

Wet ball‐milling of coarse azoxystrobin crystals in the presence of PDMAC_30_‐PCL_16_‐PDMAC_30_ nanoparticles produced a 20 % w/w aqueous suspension within 30 min at 20 °C. Optical microscopy studies confirmed a substantial reduction in mean particle size (Figures 9a and 9b) while laser diffraction studies indicated that the final azoxystrobin microparticles had a mean diameter of 2.0 μm (Figure 9c). These observations are very similar to those obtained when using non‐degradable methacrylic nanoparticles as a dispersant.[^10^, ^11^] In principle, such hydrolytically degradable nanoparticles could be used to prepare more environmentally‐friendly next‐generation agrochemical formulations.

## Conclusion

A new strategy for *reverse sequence* PISA has been developed that enables the efficient synthesis of hydrolytically degradable block copolymer nanoparticles in aqueous media. This approach involves solubilization of a trithiocarbonate‐capped monofunctional or bifunctional poly(ϵ‐caprolactone) precursor in DMAC. RAFT polymerization of this monomer is then conducted either in the bulk or as an 80 % w/w aqueous solution. At a suitable intermediate conversion, the reaction mixture is diluted by addition of deoxygenated deionized water. Thereafter, the DMAC polymerization proceeds to essentially full conversion within 16 h at 80 °C, producing a 10–25 % w/w aqueous dispersion of sterically‐stabilized PCL‐core nanoparticles. High blocking efficiencies and narrow molecular weight distributions (*M*_w_ */M*_n_≤1.30) are obtained, which indicates that the DMAC polymerization is well‐controlled. TEM analysis revealed a spherical copolymer morphology regardless of the copolymer composition or architecture, while DLS studies indicated apparent z‐average diameters ranging from 20 to 120 nm. Aging 1.0 % w/w aqueous dispersions of PDMAC_50_‐PCL_16_‐PDMAC_50_ nanoparticles at 37 °C led to extensive hydrolytic degradation at either pH 2.9 or pH 10.8. A slower rate of degradation was also observed under milder conditions (pH 7.4), whereas no discernible hydrolysisoccurred when aging the same nanoparticles for 12 weeks in deionized water (10 % w/w solids, pH 6.7) at 20 °C. Finally, PDMAC_30_‐PCL_16_‐PDMAC_30_ nanoparticles were evaluated as dispersants for the preparation of concentrated aqueous suspensions of a broad‐spectrum fungicide (azoxystrobin) via wet ball‐milling. This processing route produced azoxystrobin microparticles of approximately 2 μm diameter. Given the very high monomer conversions, narrow molecular weight distributions and aqueous formulations, *reverse sequence* PISA offers a highly convenient route to hydrolytically degradable nanoparticles. In this context, it represents an interesting alternative strategy to the statistical copolymerization of cyclic monomers with vinyl monomers, as recently reported by other research groups.

## Supporting Information

The authors have cited additional references within the Supporting Information.[^54^, ^55^] Supporting data for this study are available within the Supporting Information of this article.

## Conflict of interest

The industrial sponsor of this study (Ashland) has filed a patent application to protect the IP associated with this study.

1

## Supporting information

As a service to our authors and readers, this journal provides supporting information supplied by the authors. Such materials are peer reviewed and may be re‐organized for online delivery, but are not copy‐edited or typeset. Technical support issues arising from supporting information (other than missing files) should be addressed to the authors.

Supporting Information

## Data Availability

The data that support the findings of this study are available in the supplementary material of this article.
