# Supplementary material for: Efficient Synthesis of Hydrolytically Degradable Block Copolymer Nanoparticles via Reverse Sequence Polymerization‐Induced Self‐Assembly in Aqueous Media
Source: Angew Chem Int Ed Engl. 2023 Aug 10;62(38):e202309526. doi: 10.1002/anie.202309526 (PMC10952355; doi:10.1002/anie.202309526)
Supplement: Supplementary file 1 — Supporting Information [file ANIE-62-0-s001.pdf]

Angewandte Chemie
Eine Zeitschrift der Gesellschaft Deutscher Chemiker

Supporting Information

Efficient Synthesis of Hydrolytically Degradable Block Copolymer Nanoparticles via Reverse Sequence Polymerization-Induced Self-Assembly in Aqueous Media

*M. A. H. Farmer, O. M. Musa, S. P. Armes**

Supporting Information for

Efficient Synthesis of Hydrolytically Degradable Block Copolymer Nanoparticles via Reverse Sequence Polymerization-Induced Self-Assembly in Aqueous Media

Matthew A. H. Farmer,[a] Osama M. Musa,[b] and Steven P. Armes[a, *]

a. Dainton Building, Department of Chemistry, University of Sheffield, Sheffield, S3 7HF, UK.

b. Ashland Specialty Ingredients, 1005 US 202/206, Bridgewater, New Jersey, 08807, USA.

*Corresponding author: s.p.armes@sheffield.ac.uk

Contents

Experimental S2
- Materials S2
- Characterization Techniques S2
- Synthetic Protocols S4

Tables of Reagent Quantities and Intermediate Conversions S8
Supplementary Reaction Scheme for the Synthesis of PCL_y-$PDMAC_z$ Diblock Copolymers S11
Supplementary Characterization Data for PCL Precursors S12
Supplementary Characterization Data for (PDMAC-)PCL-PDMAC Copolymers S19
References S28

Experimental

Materials

All reagents were used as received, unless stated otherwise. 4,4′-Azobis(4-cyanopentanoic acid) (ACVA; 98%), *N,N'*-dicyclohexylcarbodiimide (DCC; 99%), *N,N′*-dimethylacrylamide (DMAC; 99%), anhydrous magnesium sulfate, lithium bromide, triazabicyclodecene (TBD), potassium dihydrogen phosphate, calcium hydride, ε-caprolactone and benzyl alcohol were purchased from Sigma-Aldrich (Dorset, UK). The latter two reagents were dried over calcium hydride and distilled before use. Ammonia solution (28 %) and 4-(dimethylamino)pyridine (DMAP) were purchased from Alfa Aesar (Heysham, UK). *N,N′*-Dimethylformamide (DMF) was purchased from VWR (Leicestershire, UK). Methanol, ammonium chloride, Oxoid™ phosphate buffered saline tablets and hydrochloric acid (38%) were purchased from Fisher Scientific (Loughborough, UK). Benzoic acid was purchased from Fluorochem Limited (Hadfield, UK). Deuterated dichloromethane (99.8%) was purchased from Goss Scientific Instruments Ltd. (Cheshire, UK). Anhydrous dichloromethane and toluene were obtained from an in-house Grubbs purification solvent system. Dihydroxy-capped poly(ε-caprolactone) was donated by Ingevity (North Charleston, South Carolina, USA). Azoxystrobin was kindly provided by Syngenta (Jealotts Hill, UK). The antifoaming agent, silicone SAG1572, was purchased from Momentive (Germany). Zirconium aluminum oxide beads of a diameter of 1.0 mm were purchased from Sigmund-Lindner (Germany). 4-Cyano-4-(ethylsulfanylthiocarbonyl)sulfanylpentanoic acid (CEPA) was prepared using a literature protocol.[52] Deionized water was dispensed from an Elgastat Option 3A water purification system with a resistivity of 15 MΩ cm.

Characterization Techniques

^{1}H Nuclear Magnetic Resonance Spectroscopy. Spectra were obtained using a 400 MHz Bruker Avance-400 spectrometer operating at 298 K with 16 scans being averaged per spectrum. Samples were dissolved in CD_2Cl_2 and aqueous dispersions of the copolymer were dried with anhydrous magnesium sulfate before passing through a 0.20 µm filter. DMAC conversions were calculated by comparing the integrated vinyl proton signals at 6.63, 6.23 and 5.66 ppm against the PCL signal at 4.08 ppm.

Gel Permeation Chromatography. An Agilent 1260 Infinity GPC system equipped with a differential refractive index detector and a UV detector set at 305 nm was used to determine the number-average molecular weight (M_n), weight-average molecular weight (M_w) and dispersity (M_w/M_n) for each (co)polymer. Two Agilent PL-gel 5 µm mixed-C columns and a guard column were connected in series to this GPC system. Unless otherwise stated, high-performance liquid chromatography (HPLC) grade DMF containing 10 mM LiBr was used as the eluent. GPC analysis was performed at 60 °C using a constant flow rate of 1.0 mL min^{-1}. A series of twelve near-monodisperse poly(methyl methacrylate) calibration standards with M_p values ranging from 800 g mol^{-1} to 2 200 000 g mol^{-1} was used to calculate molecular weights and dispersities. All (co)polymer samples were diluted to 1.0% w/w using the GPC eluent and chromatograms were analyzed using Agilent GPC/SEC software.

Dynamic Light Scattering. Unless stated otherwise, experiments were conducted at 20 °C using a Malvern Instruments Zetasizer Nano ZS instrument equipped with a 4 mW He−Ne laser (λ = 633 nm). Scattered light was detected at 173 ° with an avalanche photodiode detector. Aqueous block copolymer dispersions were diluted to 0.1% w/w with deionized water prior to analysis. Five minutes was allowed for thermal equilibration at the beginning of each measurement. The mean z-average particle diameter (D_z) and polydispersity index (PDI) were averaged over three consecutive runs consisting of ten measurements each.

Transmission Electron Microscopy (TEM). Copper/palladium grids (Agar Scientific, UK) were coated in-house with a thin film of amorphous carbon and then treated with a plasma glow discharge for 30 seconds to generate a hydrophilic surface. A 10 µL droplet of freshly diluted 0.1% w/w aqueous copolymer dispersion was placed on a hydrophilic grid for 1 min, then blotted to remove excess sample. Each grid was negatively stained for a further 25 seconds using a 10 µL droplet of 0.75% w/v aqueous uranyl formate solution, which was then carefully blotted to remove excess stain. Each grid was dried with the aid of a vacuum hose. Imaging was performed using a FEI Tecnai Spirit 2 microscope equipped with an Orius SC1000B camera operating at 80 kV.

Laser Diffraction. Laser diffraction was performed on both the initial coarse azoxystrobin crystals and the ball-milled microparticles of azoxystrobin. A Malvern Mastersizer 3000 instrument equipped with a Hydro EV wet dispersion unit set at 1500 rpm was used for such measurements. Samples were allowed to equilibrate for ten minutes before measurements. The volume-average particle diameter, d(0.5), was calculated by averaging over three measurements with an assumed absorption index of 0.10.

Optical Microscopy. Cole-Palmer optical microscope equipped with a Moticam camera was used for imaging azoxystrobin crystals before and after wet ball-milling.

Aqueous Electrophoresis. A Malvern Instruments Zetasizer Nano ZS instrument was used for electrophoretic characterization of the copolymer dispersions diluted to 0.1% w/w using 1 mM KCl as background electrolyte. Mobilities were determined at 20 °C and the solution pH adjustments were performed using either 0.1 M NaOH or 0.1 M HCl as required. Zeta potentials were calculated from the Henry equation using the Smoluchowski approximation.

Shear-induced polarized light imaging. An Anton Paar Physica MCR301 with SIPLI attachment was used for conducting shear alignment experiments. For all experiments, a plate−plate geometry comprising of a 25 mm polished steel plate and a fused quartz plate was used with a zero gap of 0.50 mm set. A Peltier system with a Peltier hood was connected to the set up to ensure good temperature control. Illumination of the samples was attained using an Edmund Optics 150 W MI-150 high-intensity fiber optic white light source. Polarized light images were obtained with the polarizer and analyzer axes crossed at 90°. Polarized light images were captured using a color CCD camera (Lumenera Lu165c).

Synthetic Protocols

Synthesis of monohydroxy-capped poly(ε-caprolactone)

The polymerization of ε-caprolactone was conducted based on a synthesis protocol reported in the literature.[53] For example, ε-caprolactone (10.77 g, 0.094 mol) was added to a flame-dried Schlenk flask charged with TBD (65.6 mg, 0.472 mmol), benzyl alcohol (0.15 g, 1.387 mmol; target DP = 68) and toluene (46 mL). This reaction mixture was stirred at 20 °C under a nitrogen atmosphere and the ensuing ring-opening polymerization was quenched after 4 h by addition of benzoic acid. The resulting solution was precipitated into excess ice-cold methanol and filtered under vacuum to afford a monohydroxy-capped poly(ε-caprolactone) with a mean DP of 42 (PCL_{42}-OH, final CL conversion = 62%). End-group analysis by ^{1}H NMR spectroscopy was used to estimate the mean degree of polymerization (DP) for each monohydroxy-capped PCL precursor by comparing the methylene unit assigned to the initiator (Ar-CH_2-O-) at 5.14 ppm to the PCL backbone signals at 4.08, 2.33, 1.66 and 1.41 ppm (see Figures S6-S8). For analogous syntheses targeting alternative PCL DPs, the monomer, catalyst, solvent and initiator quantities were adjusted accordingly (see Table S4).

Synthesis of trithiocarbonate-capped monofunctional PCL precursors

All glassware was dried in a 200 °C oven for 24 h prior to use. DMAP (0.02 g, 0.194 mmol), DCC (0.71 g, 0.003 mol), monohydroxy-capped PCL_{42} (5.00 g, 1.020 mmol) and CEPA (0.46 g, 1.733 mmol) were weighed into four separate dry 28 mL vials, sealed with rubber septa, and further dried in a vacuum oven for 2 h at 35 °C. A minimal amount of anhydrous CH_2Cl_2 was added to these four vials using a syringe/needle to dissolve each reagent. The three CH_2Cl_2 solutions containing CEPA, PCL_{42} and DMAP respectively were then transferred via syringe/needle into a 100 mL two-necked round-bottom flask fitted with a condenser, charged with a magnetic stirrer bar, and sealed with a rubber septum. This flask was then immersed in an ice bath and the CH_2Cl_2 solution containing DCC was added dropwise via syringe/needle. The reaction mixture was heated to reflux while purging with dry N_2 gas and then refluxed for 48 h. The reaction mixture was cooled to 20 °C, filtered to remove the insoluble *N,N'*-dicyclohexylurea by-product, and the solution was concentrated to approximately 2 mL under vacuum. The crude PCL_{42}-TTC was purified by precipitation into excess ice-cold methanol, filtered under vacuum and washed copiously with ice cold methanol to remove impurities. GPC analysis (using a UV detector set at 305 nm) confirmed that the purified PCL_{42}-TTC precursor contained no residual CEPA (see Figure S13). ^{1}H NMR spectroscopy was used to assess the mean degree of esterification via end-group analysis, comparing the integrated proton signal at 1.91 ppm assigned to the methyl group of the RAFT agent with the unique PCL backbone signals at 4.08, 2.33 and 1.66 ppm. For analogous syntheses in which the mean DP (21, 29 and 42) of the monofunctional PCL precursor was varied, the reagent quantities were adjusted accordingly (see Table S5).

Synthesis of the bifunctional trithiocarbonate-capped PCL precursor

All glassware was dried in a 200 °C oven for 24 h prior to use. DMAP (0.25 g, 2.022 mmol), DCC (3.72 g, 0.018 mol), dihydroxy-capped PCL_{16} (5.00 g, 2.656 mmol) and CEPA (2.37 g, 9.027 mmol) were weighed into four separate dry 28 mL vials, sealed with rubber septa, and further dried in a vacuum oven for 2 h at 35 °C. A minimal amount of anhydrous CH_2Cl_2 was added to the four vials using a syringe/needle to dissolve each reagent. The three CH_2Cl_2 solutions containing CEPA, PCL_{16} and DMAP respectively were then transferred via syringe/needle into a 100 mL two-necked round-bottom flask fitted with a condenser, charged with a magnetic stirrer bar, and sealed with a rubber septum. This flask was then immersed in an ice bath and the CH_2Cl_2 solution

containing DCC was added dropwise via syringe/needle. The reaction mixture was heated to reflux while purging with dry N_2 gas and then refluxed for 48 h. The reaction mixture was cooled to 20 °C, filtered to remove the insoluble *N,N'*-dicyclohexylurea by-product, and the solution was concentrated to approximately 2 mL under vacuum. The crude TTC-PCL$_{16}$-TTC was purified by precipitation into excess ice-cold methanol, filtered under vacuum and washed copiously with methanol to remove impurities. GPC analysis (using a UV detector set at 305 nm) confirmed that the purified TTC-PCL$_{16}$-TTC precursor contained no residual CEPA (see Figure S13). ^{1}H NMR spectroscopy was used to assess the mean degree of esterification via end-group analysis, comparing the integrated proton signal at 1.91 ppm assigned to the methyl group of the RAFT agent with the unique PCL backbone signals at 4.08, 2.33 and 1.66 ppm.

RAFT polymerization of DMAC in the bulk using TTC-PCL$_{16}$-TTC with subsequent dilution with water at an intermediate DMAC conversion

A 28 mL vial was charged with TTC-PCL$_{16}$-TTC (0.10 g, 0.043 mmol), DMAC (0.68 g, 6.869 mmol, target DP = 80), ACVA (4.8 mg, 0.017 mmol, [TTC]/[ACVA] molar ratio = 5.0) and a magnetic stirrer bar and sealed with a rubber septum. This vial was placed in an ice bath and deoxygenated with a stream of dry N_2 gas for 30 min. The vial was then allowed to warm to room temperature for 10 min before being immersed in an oil bath set at 80 °C. The reaction mixture was stirred magnetically and monitored by visual inspection. As soon as the reaction mixture became much more viscous after 7.5 min, deoxygenated deionized water (7.07 mL, preheated to 80 °C, targeting 10% w/w solids) was added using a degassed syringe/needle. At this point, the reaction vial was removed from the oil bath and subjected to vortex mixing for 2 min to ensure a homogeneous solution, then reimmersed in the oil bath. At this time point, the reaction mixture was sampled and ^{1}H NMR spectroscopy analysis indicated an instantaneous DMAC conversion of 37% (PDMAC DP ~ 30). The DMAC polymerization was allowed to proceed for 16 h prior to quenching by exposing the reaction mixture to air while cooling to 20 °C. Occasionally, a small amount of gel formed at the top of the vial but this could be redispersed into the solution by vortex mixing. A final DMAC conversion of more than 99% was indicated by ^{1}H NMR studies. For analogous syntheses in which the initial solids content, target PDMAC DP and both type and nature of the PCL-based RAFT agent were varied, the reagent quantities and volume of added water were adjusted accordingly (see Tables S1 and S3).

RAFT polymerization of DMAC in the bulk using PCL$_{42}$-TTC with subsequent dilution with water at an intermediate DMAC conversion

A 28 mL vial was charged with PCL$_{42}$-TTC (0.10 g, 0.019 mmol), DMAC (0.23 g, 2.324 mmol, target DP = 120), ACVA (1.1 mg, 0.004 mmol, [TTC]/[ACVA] molar ratio = 5.0) and a magnetic stirrer bar and sealed with a rubber septum. This vial was placed in an ice bath and deoxygenated with a stream of dry N_2 gas for 30 min. The vial was then allowed to warm to room temperature for 10 min before being immersed in an oil bath set at 80 °C. The reaction mixture was stirred magnetically and monitored by visual inspection. As soon as the reaction mixture became much more viscous after 14 min, deoxygenated deionized water (2.98 mL, preheated to 80 °C, targeting 10% w/w solids) was added using a degassed syringe/needle. At this point, the reaction vial was removed from the oil bath and subjected to vortex mixing for 2 min to ensure a homogeneous solution, then reimmersed in the oil bath. At this time point, the reaction mixture was sampled and ^{1}H NMR spectroscopy analysis indicated an instantaneous DMAC conversion of 60% (PDMAC DP ~ 72). The DMAC polymerization was allowed to proceed for 16 h prior to quenching by exposing the reaction mixture to air while cooling to 20 °C. Occasionally, a small amount of gel formed at the top of the vial but this could be redispersed into the solution by vortex mixing. A final DMAC conversion of more than 99% was indicated by ^{1}H NMR studies. For analogous syntheses

in which the initial solids content, target PDMAC DP and both type and nature of the PCL-based RAFT agent were varied, the reagent quantities and volume of added water were adjusted accordingly (see Table S1).

RAFT polymerization of DMAC in 80% w/w solids aqueous solution using TTC-PCL$_{16}$-TTC with subsequent dilution with water at an intermediate DMAC conversion

A 28 mL vial was charged with TTC-PCL$_{16}$-TTC (0.10 g, 0.043 mmol), DMAC (0.68 g, 6.869 mmol, target DP = 80), ACVA (4.8 mg, 0.017 mmol, [TTC]/[ACVA] molar ratio = 5.0), deionized water (0.20 mL, 80% w/w solids) and a magnetic stirrer bar and sealed with a rubber septum. This vial was placed in an ice bath and deoxygenated with a stream of dry N_2 gas for 30 min. The vial was then allowed to warm to room temperature for 10 min before being immersed in an oil bath set at 80 °C. The reaction mixture was stirred magnetically and monitored by visual inspection. As soon as the reaction mixture became much more viscous after 10min, deoxygenated deionized water (6.87 mL, preheated to 80 °C, targeting 10% w/w solids) was added using a degassed syringe/needle. At this point, the reaction vial was removed from the oil bath and subjected to vortex mixing for 2 min to ensure a homogeneous solution, then reimmersed in the oil bath. At this time point, the reaction mixture was sampled and ^{1}H NMR spectroscopy analysis indicated an instantaneous DMAC conversion of 59% (PDMAC DP ~ 47). The DMAC polymerization was allowed to proceed for 16 h prior to quenching by exposing the reaction mixture to air while cooling to 20 °C. Occasionally, a small amount of gel formed at the top of the vial but this could be redispersed into the solution by vortex mixing. A final DMAC conversion of more than 99% was indicated by ^{1}H NMR studies. For analogous syntheses in which the initial solids content, target PDMAC DP and both type and nature of the PCL-based RAFT agent were varied, the reagent quantities and volume of added water were adjusted accordingly (see Table S2).

RAFT polymerization of DMAC in 80% w/w solids aqueous solution using PCL$_{42}$-TTC with subsequent dilution with water at an intermediate DMAC conversion

A 28 mL vial was charged with PCL$_{42}$-TTC (0.10 g, 0.019 mmol), DMAC (0.23 g, 2.324 mmol, target DP = 120), ACVA (1.1 mg, 0.004 mmol, [TTC]/[ACVA] molar ratio = 5.0), deionized water (0.08 mL, 80% w/w solids) and a magnetic stirrer bar and sealed with a rubber septum. This vial was placed in an ice bath and deoxygenated with a stream of dry N_2 gas for 30 min. The vial was then allowed to warm to room temperature for 10 min before being immersed in an oil bath set at 80 °C. The reaction mixture was stirred magnetically and monitored by visual inspection. As soon as the reaction mixture became much more viscous after 7 min, deoxygenated deionized water (2.90 mL, preheated to 80 °C, targeting 10% w/w solids) was added using a degassed syringe/needle. At this point, the reaction vial was removed from the oil bath and subjected to vortex mixing for 2 min to ensure a homogeneous solution, then reimmersed in the oil bath. At this time point, the reaction mixture was sampled and ^{1}H NMR spectroscopy analysis indicated an instantaneous DMAC conversion of 21% (PDMAC DP ~ 25). The DMAC polymerization was allowed to proceed for 16 h prior to quenching by exposing the reaction mixture to air while cooling to 20 °C. A final DMAC conversion of more than 99% was indicated by ^{1}H NMR studies. For analogous syntheses in which the initial solids content, target PDMAC DP and both type and nature of the PCL-based RAFT agent were varied, the reagent quantities and volume of added water were adjusted accordingly (see Table S2).

Hydrolytic degradation of block copolymers in aqueous solution

A series of aqueous solutions were prepared as follows. Potassium dihydrogen phosphate (10.00 g, 0.073 mol) was dissolved in deionized water (80 mL). Then the solution pH was adjusted with 0.1 M HCl and made up to 100 mL using further deionized water to provide a final solution pH of 2.9. Ammonium chloride (6.80 g, 0.127 mol) was dissolved in 28% aqueous ammonia solution (100 mL) to afford a final solution pH of 10.8. A single Oxoid PBS tablet was dissolved in deionized water (100 mL) and 0.1 M HCl was used to adjust the solution pH to pH 7.4. A 10% w/w aqueous dispersion of $PDMAC_{50}$-PCL_{16}-$PDMAC_{50}$ nanoparticles was diluted to 1.0% w/w using each of the above aqueous solutions in turn. The resulting three aqueous dispersions were stirred at 37 ^{o}C for four weeks and sampled periodically for GPC analysis. The same protocol was employed to study the hydrolytic degradation of aqueous dispersions of PCL_{21}-$PDMAC_{70}$ and PCL_{42}-$PDMAC_{120}$ nanoparticles.

Preparation of Azoxystrobin Suspension Concentrates by Ball Milling

Azoxystrobin (2.00 g), $PDMAC_{30}$-PCL_{16}-$PDMAC_{30}$ nanoparticles (0.25 g, 10% w/w), SAG1572 antifoam (0.10 g, 1.0% w/w) and deionized water (7.65 g) were added to a 30 mL sample tube containing 1.0 mm ceramic beads (10.00 g). This aqueous suspension was then ball-milled using an IKA Ultra-Turrax Tube Drive at 6 000 rpm for 30 min. The beads were removed by filtration to afford a 20% w/w aqueous suspension of azoxystrobin microparticles. This suspension was purified by centrifugation for 10 min at 13 000 rpm using a Thermo Heraeus Biofuge Pico centrifuge. The aqueous supernatant was decanted and the sedimented azoxystrobin microparticles were redispersed in deionized water. Two further centrifugation-redispersion cycles were performed to remove any excess non-adsorbed triblock copolymer nanoparticles prior to characterization by optical microscopy and laser diffraction.

Tables of Reagent Quantities and Intermediate Conversions

Table S1. Summary of reagent quantities used for the initial bulk polymerization of DMAC using either a TTC-PCL_{16}-TTC or a PCL_y-TTC precursor, with subsequent dilution to 10% w/w solids via addition of deoxygenated deionized water. Instantaneous conversions at the time of dilution are determined via ^1H NMR spectroscopy.

Entry Number	Polymer composition	(TTC-)PCL-TTC	DMAC	ACVA	Water	Dilution Time	Intermediate conversion
1	PD_{80}-PCL_{16}-PD_{80}	0.10 g (0.043 mmol)	0.68 g (6.869 mmol)	4.8 mg (0.017 mmol)	7.07 mL	7.5 min	37%
2	PD_{70}-PCL_{16}-PD_{70}	0.10 g (0.043 mmol)	0.60 g (6.011 mmol)	4.8 mg (0.017 mmol)	6.31 mL	8 min	59%
3	PD_{60}-PCL_{16}-PD_{60}	0.10 g (0.043 mmol)	0.51 g (5.152 mmol)	4.8 mg (0.017 mmol)	5.54 mL	9 min	60%
4	PD_{50}-PCL_{16}-PD_{50}	0.10 g (0.043 mmol)	0.43 g (4.293 mmol)	4.8 mg (0.017 mmol)	4.77 mL	9 min	33%
5	PD_{40}-PCL_{16}-PD_{40}	0.10 g (0.043 mmol)	0.34 g (3.435 mmol)	4.8 mg (0.017 mmol)	4.01 mL	9 min	60%
6	PD_{30}-PCL_{16}-PD_{30}	0.10 g (0.043 mmol)	0.26 g (2.576 mmol)	4.8 mg (0.017 mmol)	3.24 mL	9.5 min	37%
7	PD_{20}-PCL_{16}-PD_{20}	0.10 g (0.043 mmol)	0.17 g (1.717 mmol)	4.8 mg (0.017 mmol)	2.48 mL	14.5min	Precipitated
8	PCL_{21}-PD_{90}	0.10 g (0.036 mmol)	0.32 g (3.253 mmol)	2.0 mg (0.007 mmol)	3.82 mL	11 min	41%
9	PCL_{21}-PD_{80}	0.10 g (0.036 mmol)	0.29 g (2.891 mmol)	2.0 mg (0.007 mmol)	3.50 mL	12 min	49%
10	PCL_{21}-PD_{70}	0.10 g (0.036 mmol)	0.25 g (2.530 mmol)	2.0 mg (0.007 mmol)	3.18 mL	12 min	53%
11	PCL_{21}-PD_{60}	0.10 g (0.036 mmol)	0.21 g (2.168 mmol)	2.0 mg (0.007 mmol)	2.85 mL	13 min	51%
12	PCL_{21}-PD_{50}	0.10 g (0.036 mmol)	0.18 g (1.807 mmol)	2.0 mg (0.007 mmol)	2.53 mL	10.5 min	26%
13	PCL_{21}-PD_{40}	0.10 g (0.036 mmol)	0.14 g (1.446 mmol)	2.0 mg (0.007 mmol)	2.21 mL	16.5 min	Precipitated
14	PCL_{29}-PD_{100}	0.10 g (0.027 mmol)	0.27 g (2.717 mmol)	1.5 mg (0.005 mmol)	3.34 mL	9.5 min	54%
15	PCL_{29}-PD_{90}	0.10 g (0.027 mmol)	0.24 g (2.446 mmol)	1.5 mg (0.005 mmol)	3.10 mL	11 min	45%
16	PCL_{29}-PD_{80}	0.10 g (0.027 mmol)	0.22 g (2.174 mmol)	1.5 mg (0.005 mmol)	2.85 mL	13 min	54%
17	PCL_{29}-PD_{70}	0.10 g (0.027 mmol)	0.19 g (1.902 mmol)	1.5 mg (0.005 mmol)	2.61 mL	11.5 min	44%
18	PCL_{29}-PD_{60}	0.10 g (0.027 mmol)	0.16 g (1.630 mmol)	1.5 mg (0.005 mmol)	2.37 mL	14.5 min	39%
19	PCL_{29}-PD_{50}	0.10 g (0.027 mmol)	0.13 g (1.359 mmol)	1.5 mg (0.005 mmol)	2.13 mL	17 min	Precipitated
20	PCL_{42}-PD_{120}	0.10 g (0.019 mmol)	0.23 g (2.324 mmol)	1.1 mg (0.004 mmol)	2.98 mL	14 min	60%
21	PCL_{42}-PD_{110}	0.10 g (0.019 mmol)	0.21 g (2.130 mmol)	1.1 mg (0.004 mmol)	2.81 mL	10 min	47%
22	PCL_{42}-PD_{100}	0.10 g (0.019 mmol)	0.19 g (1.936 mmol)	1.1 mg (0.004 mmol)	2.64 mL	12 min	52%
23	PCL_{42}-PD_{90}	0.10 g (0.019 mmol)	0.17 g (1.743 mmol)	1.1 mg (0.004 mmol)	2.46 mL	13 min	60%
24	PCL_{42}-PD_{80}	0.10 g (0.019 mmol)	0.15 g (1.549 mmol)	1.1 mg (0.004 mmol)	2.29 mL	10 min	29%
25	PCL_{42}-PD_{70}	0.10 g (0.019 mmol)	0.13 g (1.356 mmol)	1.1 mg (0.004 mmol)	2.12 mL	20 min	Precipitated

Table S2. Summary of reagent quantities used for the initial high solids polymerization of DMAC using either a TTC-PCL_{16}-TTC or a PCL_y-TTC precursor, with subsequent dilution via addition of deoxygenated deionized water. Instantaneous conversions at the time of dilution are determined via ^{1}H NMR spectroscopy.

Entry Number	Polymer composition	(TTC-)PCL-TTC	DMAC	ACVA	Total water (Initial water)	Dilution Time	Intermediate conversion
1	PD_{80}-PCL_{16}-PD_{80}	0.10 g (0.043 mmol)	0.68 g (6.869 mmol)	4.8 mg (0.017 mmol)	7.07 mL (0.20 mL)	10 min	59%
2	PD_{70}-PCL_{16}-PD_{70}	0.10 g (0.043 mmol)	0.60 g (6.011 mmol)	4.8 mg (0.017 mmol)	6.31 mL (0.18 mL)	10 min	59%
3	PD_{60}-PCL_{16}-PD_{60}	0.10 g (0.043 mmol)	0.51 g (5.152 mmol)	4.8 mg (0.017 mmol)	5.54 mL (0.15 mL)	9 min	57%
4	PD_{50}-PCL_{16}-PD_{50}	0.10 g (0.043 mmol)	0.43 g (4.293 mmol)	4.8 mg (0.017 mmol)	4.77 mL (0.13 mL)	9 min	52%
5	PD_{40}-PCL_{16}-PD_{40}	0.10 g (0.043 mmol)	0.34 g (3.435 mmol)	4.8 mg (0.017 mmol)	4.01 mL (0.11 mL)	9 min	52%
6	PD_{30}-PCL_{16}-PD_{30}	0.10 g (0.043 mmol)	0.26 g (2.576 mmol)	4.8 mg (0.017 mmol)	3.24 mL (0.09 mL)	13 min	60%
7	PCL_{21}-PD_{80}	0.10 g (0.036 mmol)	0.29 g (2.891 mmol)	2.0 mg (0.007 mmol)	3.50 mL (0.10 mL)	10.5 min	34%
8	PCL_{29}-PD_{100}	0.10 g (0.027 mmol)	0.27 g (2.717 mmol)	1.5 mg (0.005 mmol)	3.34 mL (0.09 mL)	12 min	45%
9	PCL_{42}-PD_{120}	0.10 g (0.019 mmol)	0.23 g (2.324 mmol)	1.1 mg (0.004 mmol)	2.98 mL (0.08 mL)	7 min	21%

Table S3. Summary of reagent quantities used for the initial bulk polymerization of DMAC using a TTC-PCL_{16}-TTC precursor, with subsequent dilution to the stated final nanoparticle concentration via addition of deoxygenated deionized water. Instantaneous conversions at the time of dilution are determined via ^{1}H NMR spectroscopy.

Entry Number	Polymer composition	(TTC-)PCL-TTC	DMAC	ACVA	Water	Dilution Time	Intermediate conversion
1	PD_{80}-PCL_{16}-PD_{80}	0.10 g (0.043 mmol)	0.68 g (6.869 mmol)	4.8 mg (0.017 mmol)	7.07 mL	7.5 min	37%
2	PD_{80}-PCL_{16}-PD_{80}	0.10 g (0.043 mmol)	0.68 g (6.869 mmol)	4.8 mg (0.017 mmol)	4.45 mL	7.5 min	31%
3	PD_{80}-PCL_{16}-PD_{80}	0.10 g (0.043 mmol)	0.68 g (6.869 mmol)	4.8 mg (0.017 mmol)	3.14 mL	7 min	46%
4	PD_{80}-PCL_{16}-PD_{80}	0.10 g (0.043 mmol)	0.68 g (6.869 mmol)	4.8 mg (0.017 mmol)	2.34 mL	7.5 min	60%

Table S4. Summary of reagent quantities used for the ring-opening polymerization of ε-caprolactone in dry toluene at 20 ˚C. Each reaction was quenched after the stated reaction time using benzoic acid.

Entry Number	Polymer Composition	Benzyl Alcohol	ε-Caprolactone	TBD	Toluene	Quench Time	Final CL Conversion
1	PCL_{21}-OH	0.15 g (1.387 mmol)	4.43 g (0.038 mol)	27.0 mg (0.194 mmol)	19 mL	3 h	75%
2	PCL_{29}-OH	0.15 g (1.387 mmol)	7.76 g (0.068 mol)	47.3 mg (0.340 mmol)	33 mL	3 h	59 %
3	PCL_{42}-OH	0.15 g (1.387 mmol)	10.77 g (0.094 mol)	65.6 mg (0.472 mmol)	46 mL	4 h	62 %

Table S5. Summary of reagent quantities used for the DCC/DMAP catalyzed esterification of poly(ε-caprolactone) precursors using the CEPA RAFT agent. Esterification was performed under nitrogen in refluxing dry CH_2Cl_2.

Entry Number	PCL Precursor	PCL	CEPA	DMAP	DCC
1	HO-PCL_{16}-OH	5.00 g (2.656 mmol)	2.37 g (9.027 mmol)	0.25 g (2.022 mmol)	3.72 g (0.018 mol)
2	PCL_{21}-OH	5.00 g (1.996 mmol)	0.89 g (3.391 mmol)	0.05 g (0.380 mmol)	1.40 g (0.006 mol)
3	PCL_{29}-OH	5.00 g (1.463 mmol)	0.63 g (2.485 mmol)	0.03 g (0.278 mmol)	0.99 g (0.005 mol)
4	PCL_{42}-OH	5.00 g (1.020 mmol)	0.46 g (1.733 mmol)	0.02 g (0.194 mmol)	0.71 g (0.003 mol)

Supplementary Reaction Scheme for the Synthesis of PCL_y-$PDMAC_z$ Diblock Copolymers

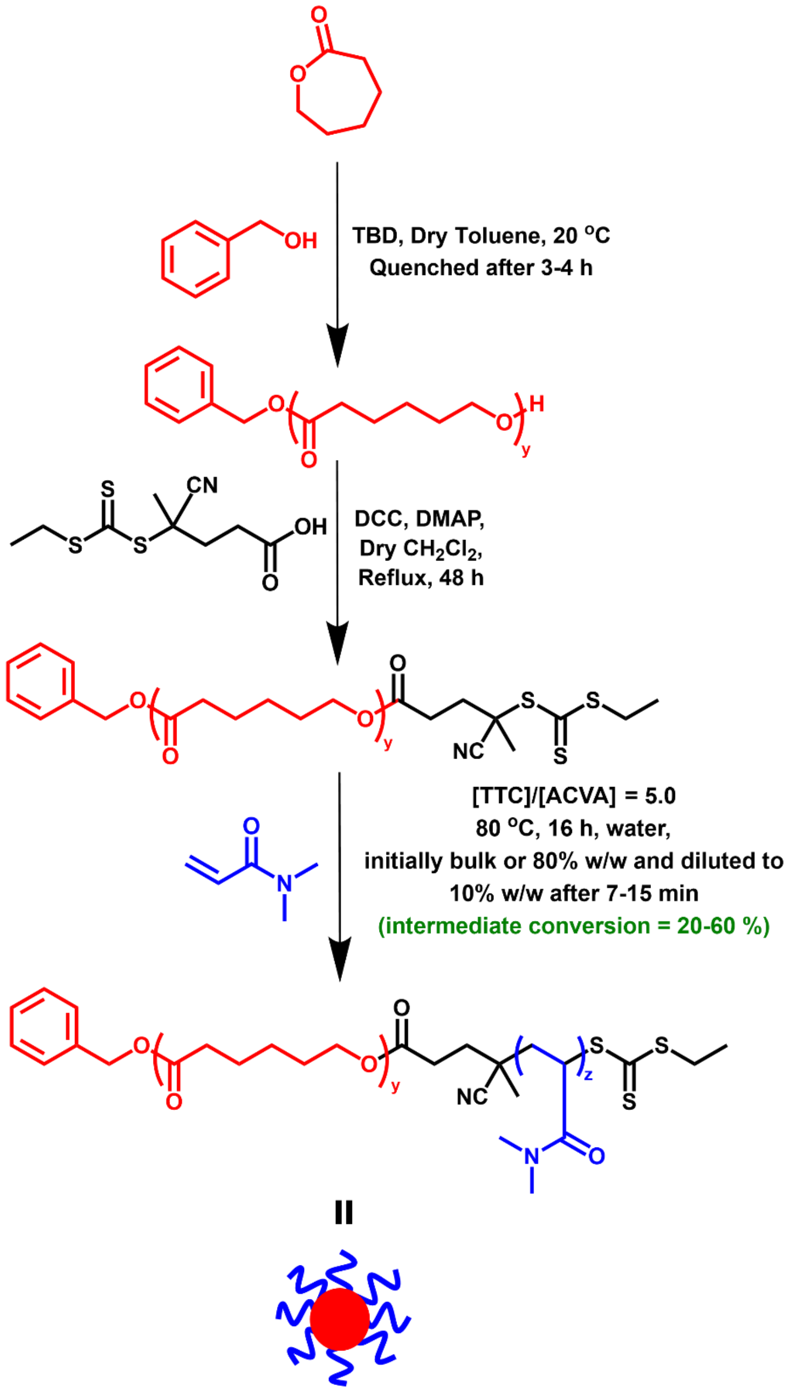

Scheme S1. Synthesis of monohydroxy-capped PCL-OH via ring-opening polymerization of ε-caprolactone in dry toluene using benzyl alcohol and TBD as a catalyst. Next, a monofunctional trithiocarbonate-capped RAFT agent (PCL_y-TTC) is prepared via DCC/DMAP-catalyzed esterification of this monofunctional PCL precursor using a carboxylic acid-functionalized RAFT agent (CEPA). Subsequently, PCL_y-$PDMAC_z$ nanoparticles are prepared at 80 °C via *reverse sequence* PISA. Initially, the RAFT polymerization of DMAC is conducted either in the bulk or in an 80% w/w aqueous solution, with subsequent dilution to 10% w/w solids using deoxygenated deionized water at a suitable intermediate DMAC conversion. Conditions: [TTC]/[ACVA] molar ratio = 5.0.

Supplementary Characterization Data for PCL Precursors

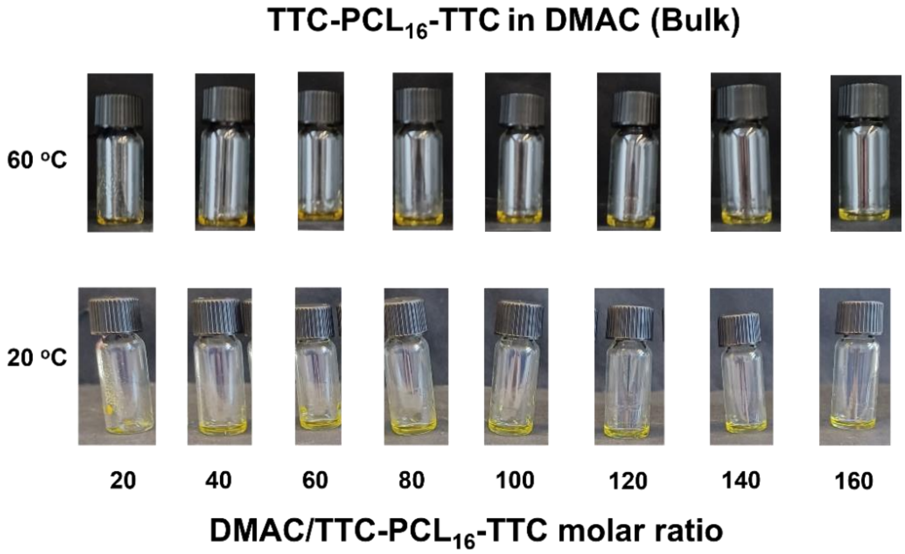

Figure S1. Digital photographs recorded for a TTC-PCL_{16}-TTC precursor after its attempted dissolution in the bulk using varying amounts of DMAC at either 60 °C or 20 °C.

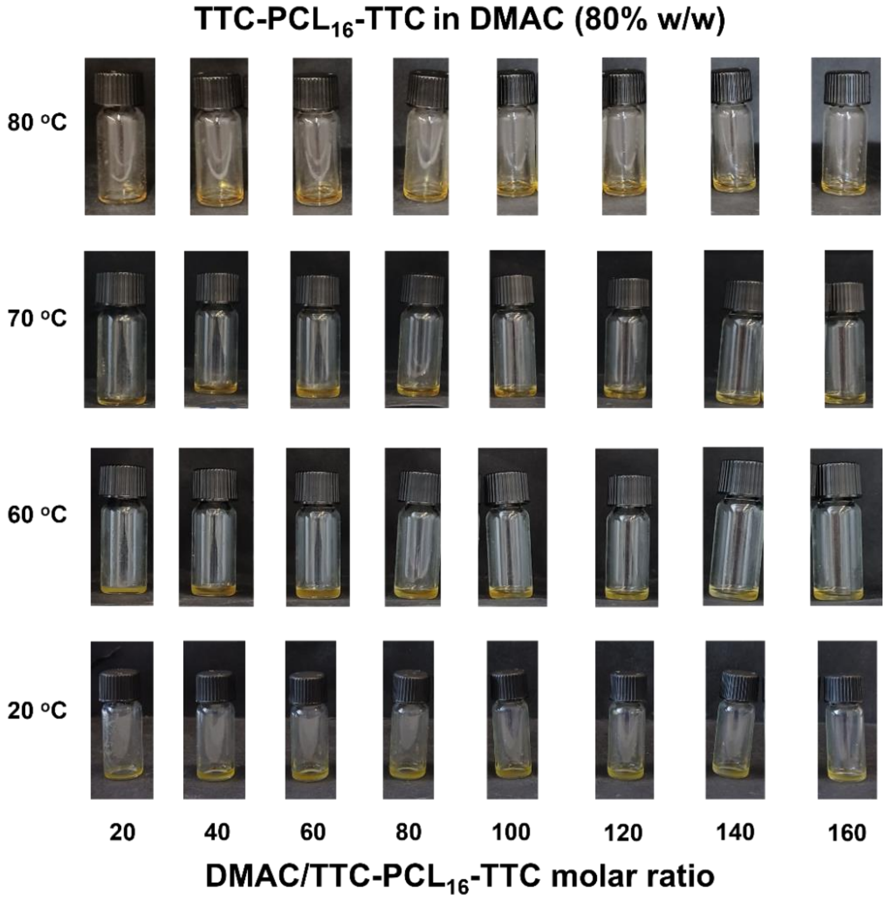

Figure S2. Digital photographs recorded for a TTC-PCL_{16}-TTC precursor after its attempted dissolution in 80% w/w aqueous solution using varying amounts of DMAC at 80 °C, 70 °C, 60 °C or 20 °C.

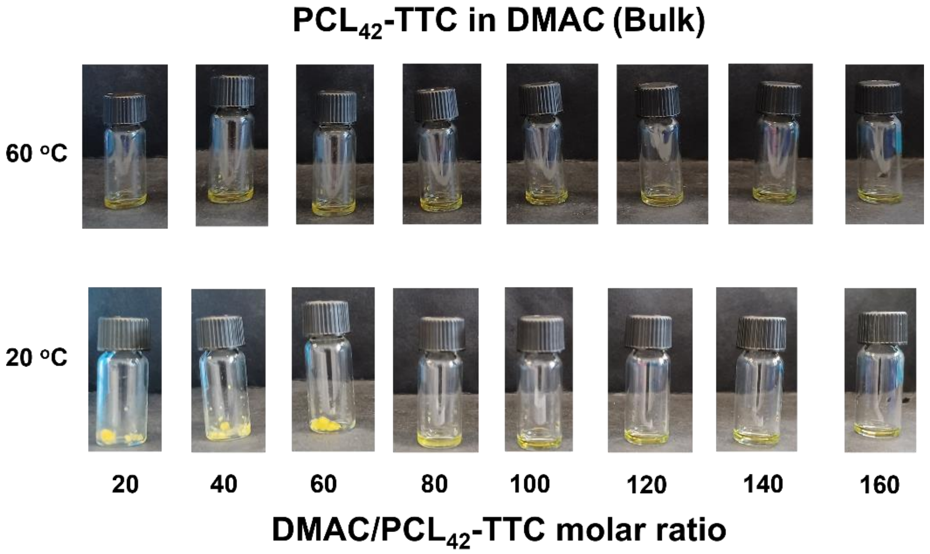

Figure S3. Digital photographs recorded for a PCL_{42}-TTC precursor after its attempted dissolution in the bulk using varying amounts of DMAC at either 60 °C or 20 °C.

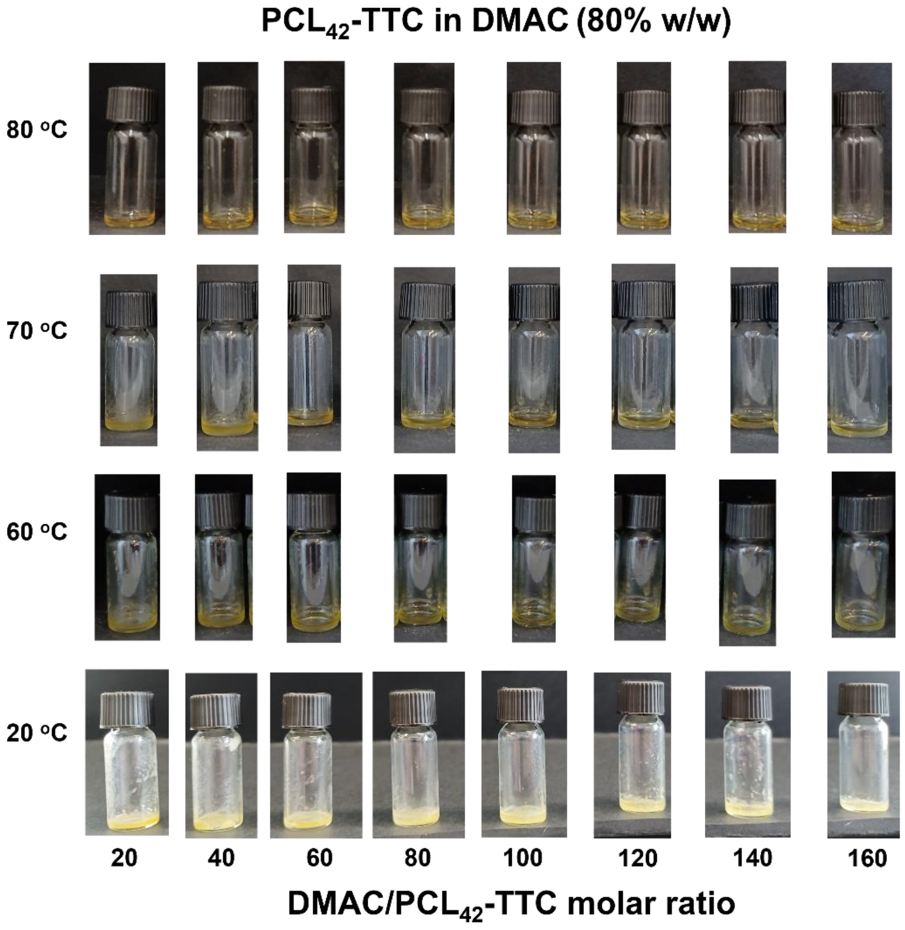

Figure S4. Digital photographs for a PCL_{42}-TTC precursor after its attempted dissolution in 80% w/w aqueous solution using varying amounts of DMAC at 80 °C, 70 °C, 60 °C or 20 °C.

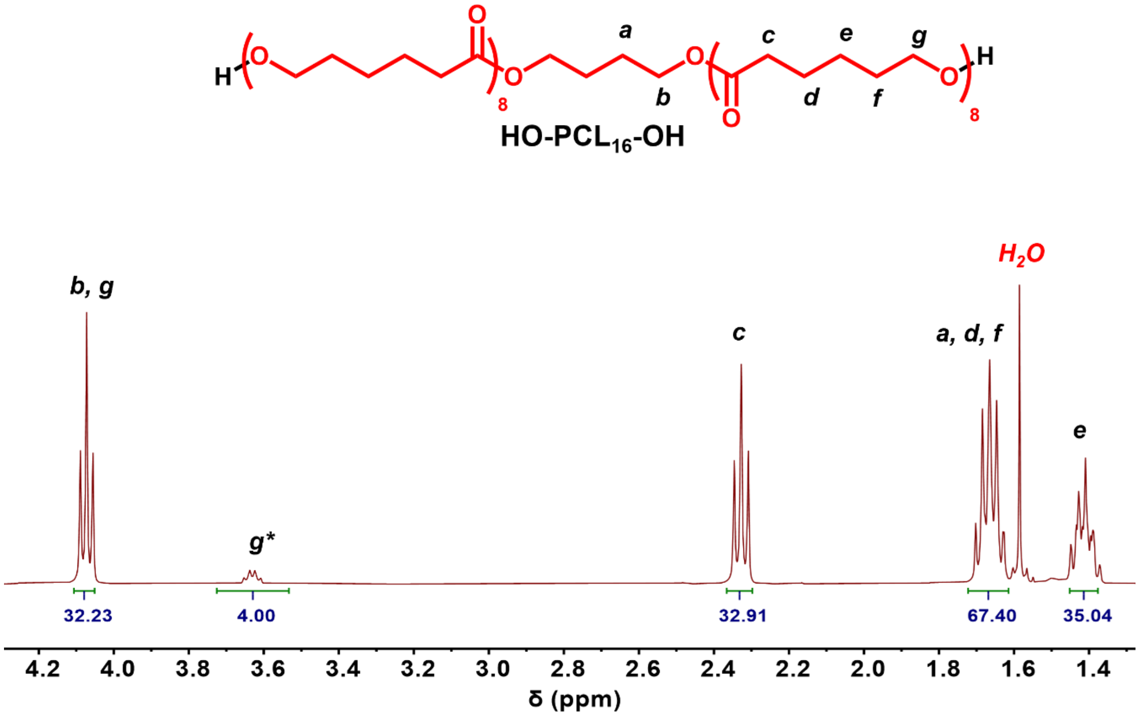

Figure S5. Assigned ^{1}H NMR spectrum (CD_2Cl_2) of the as-received HO-PCL$_{16}$-OH precursor (where g* represents the terminal g protons; mean degree of polymerization 16.4 ± 0.6).

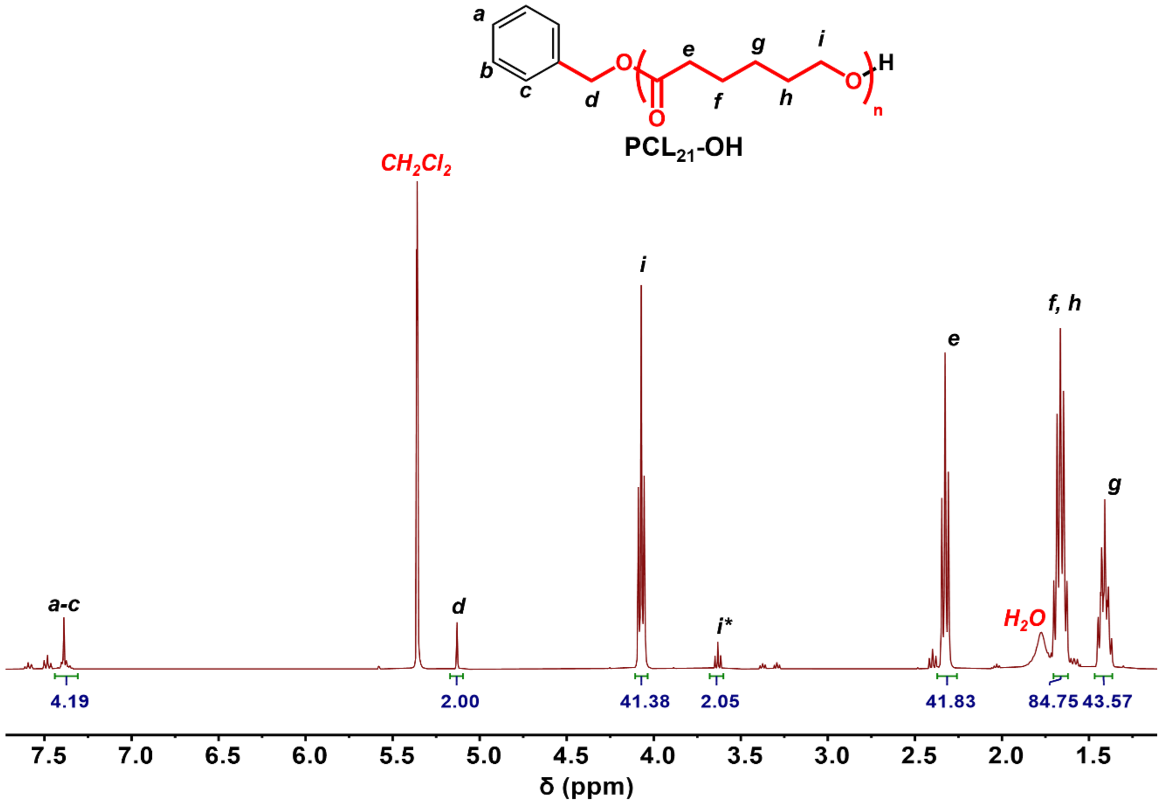

Figure S6. Assigned ^{1}H NMR spectrum (CD_2Cl_2) for a PCL$_{21}$-OH precursor prepared via ring-opening polymerization of ε-caprolactone in dry toluene (where i* represents the terminal i protons; mean degree of polymerization 21.4 ± 0.4).

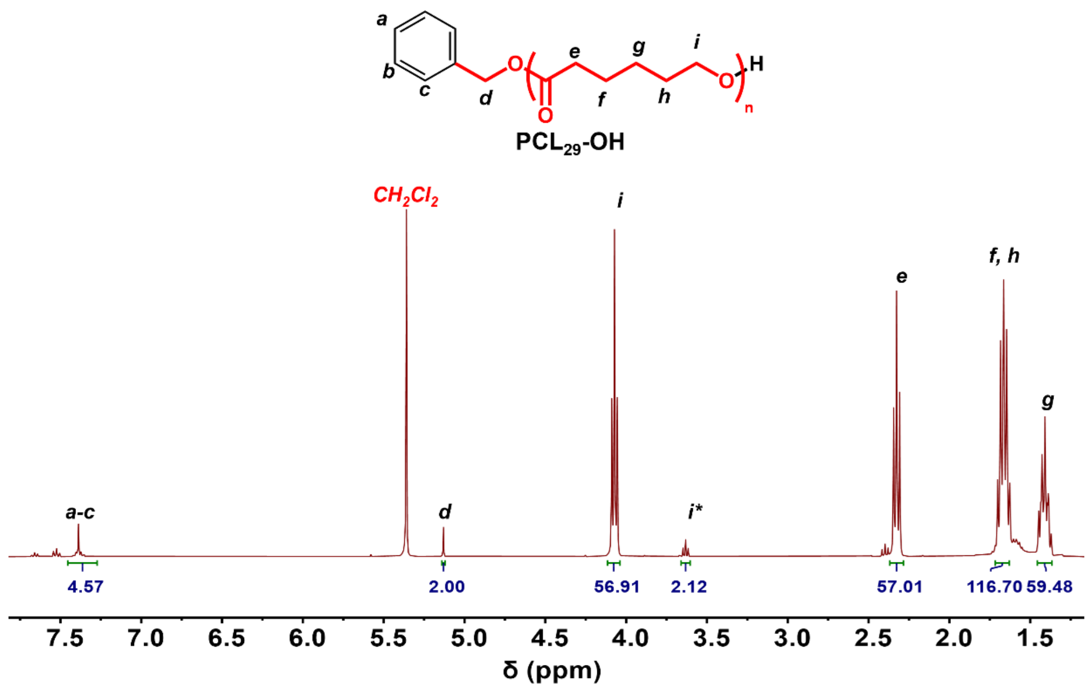

Figure S7. Assigned ^{1}H NMR spectrum (CD_2Cl_2) for a PCL_{29}-OH precursor prepared via ring-opening polymerization of ε-caprolactone in dry toluene (where i* represents the terminal i protons; mean degree of polymerization 29.2 ± 0.5).

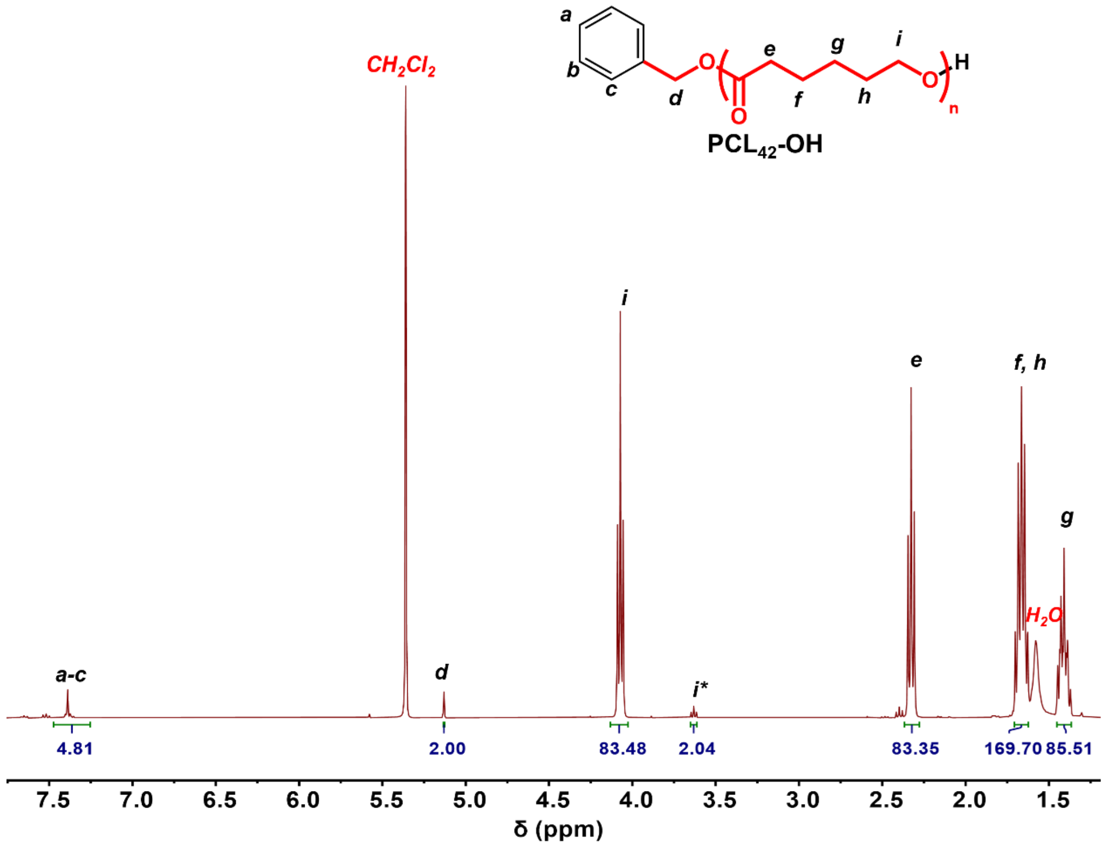

Figure S8. Assigned ^{1}H NMR spectrum (CD_2Cl_2) for a PCL_{42}-OH precursor prepared via ring-opening polymerization of ε-caprolactone in dry toluene (where i* represents the terminal i protons; mean degree of polymerization 42.4 ± 0.4).

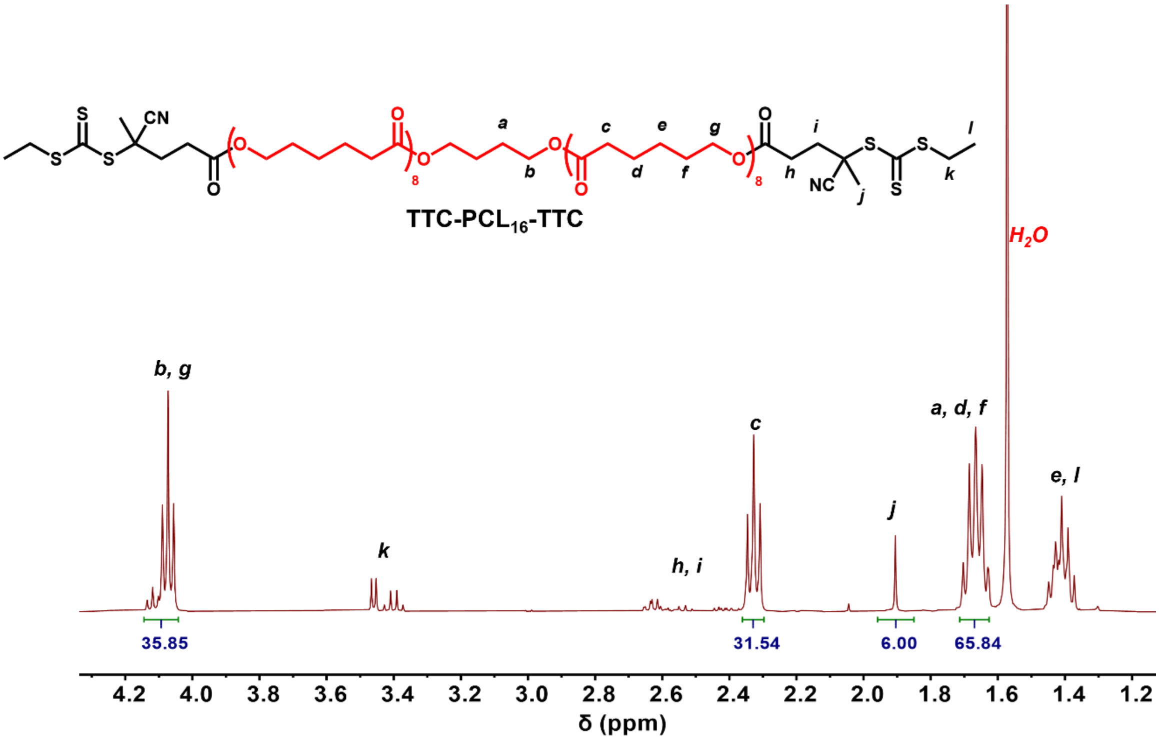

Figure S9. Assigned ^{1}H NMR spectrum (CD_2Cl_2) recorded for TTC-PCL_{16}-TTC, which was prepared via DCC/DMAP-catalyzed esterification of a HO-PCL_{16}-OH precursor using a carboxylic-functionalized RAFT agent (CEPA) (mean degree of esterification 98 ± 1%).

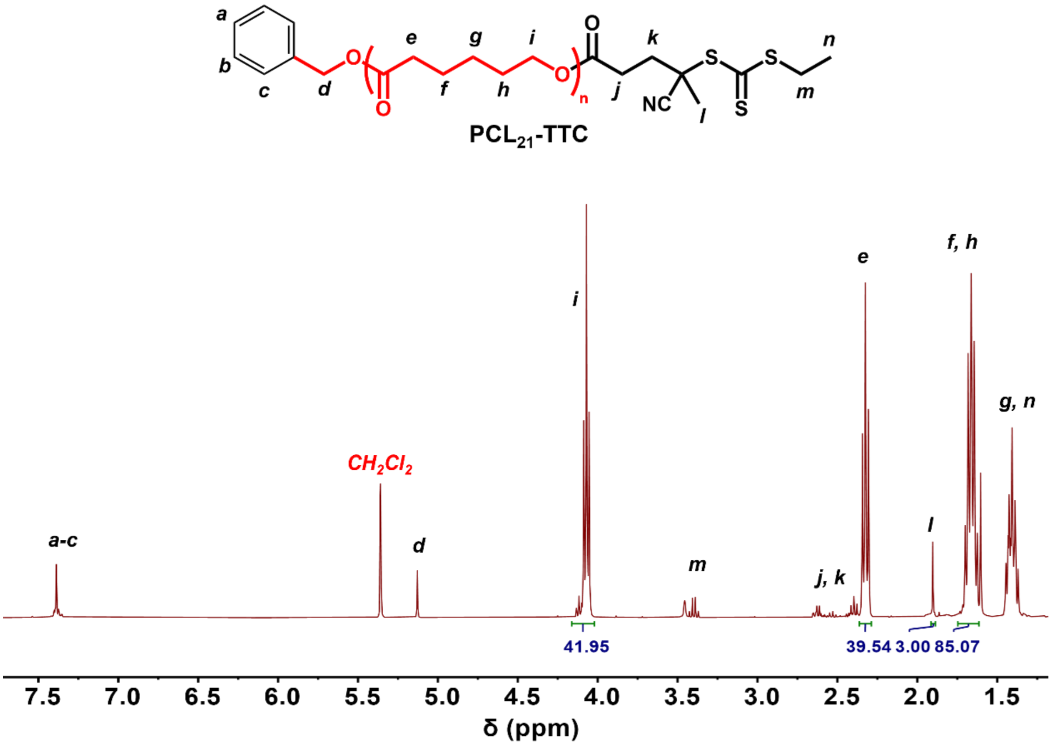

Figure S10. Assigned ^{1}H NMR spectrum (CD_2Cl_2) recorded for PCL_{21}-TTC, which was prepared via DCC/DMAP-catalyzed esterification of a PCL_{21}-OH precursor using a carboxylic-functionalized RAFT agent (CEPA) (mean degree of esterification 98 ± 3%).

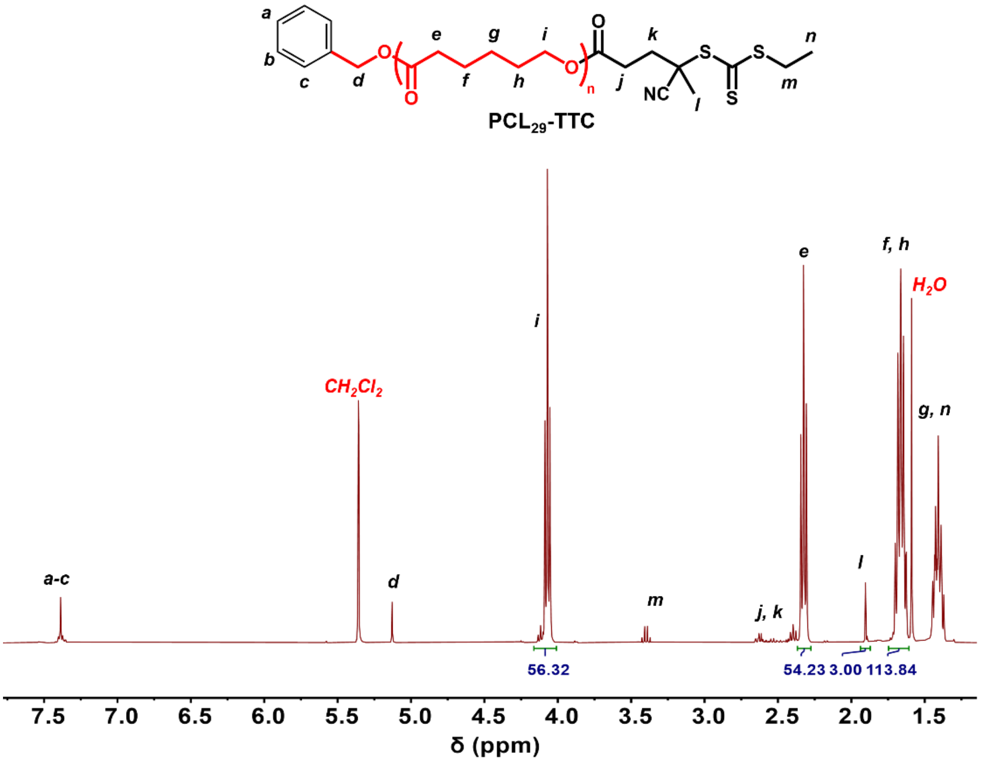

Figure S11. Assigned ^{1}H NMR spectrum (CD_2Cl_2) recorded for PCL_{29}-TTC, which was prepared via DCC/DMAP-catalyzed esterification of a PCL_{29}-OH precursor using a carboxylic acid-functionalized RAFT agent (CEPA) (mean degree of esterification 96 ± 2%).

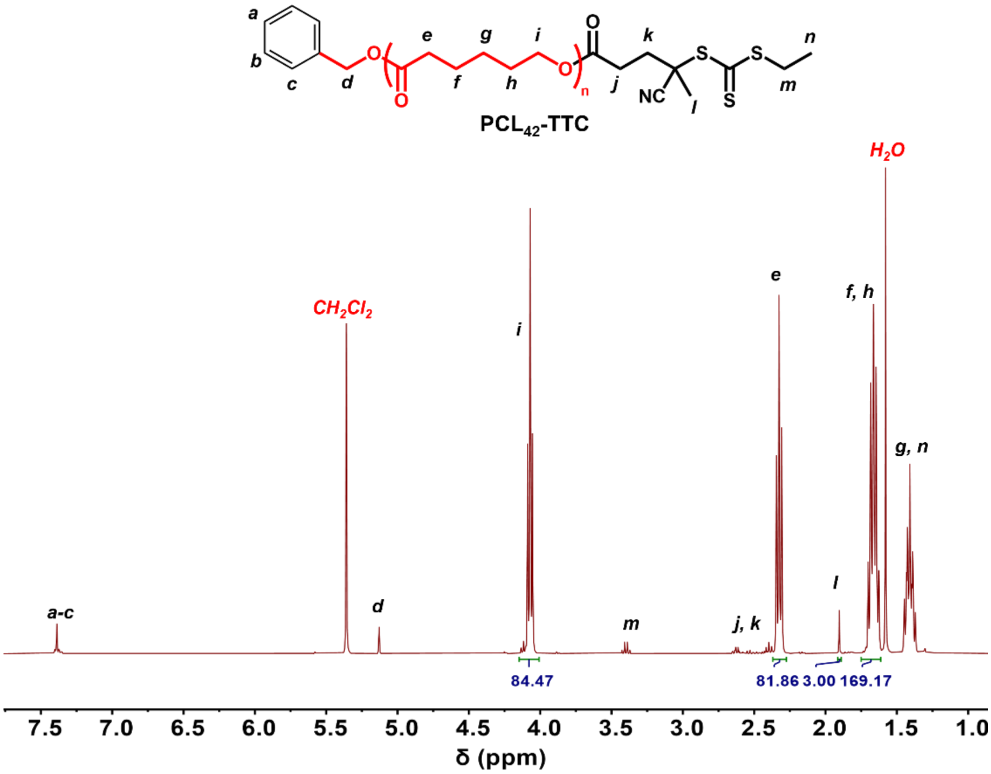

Figure S12. Assigned ^{1}H NMR spectrum (CD_2Cl_2) recorded for PCL_{42}-TTC, which was prepared via DCC/DMAP-catalyzed esterification of a PCL_{42}-OH precursor using a carboxylic acid-functionalized RAFT agent (CEPA) (mean degree of esterification 100 ± 1%).

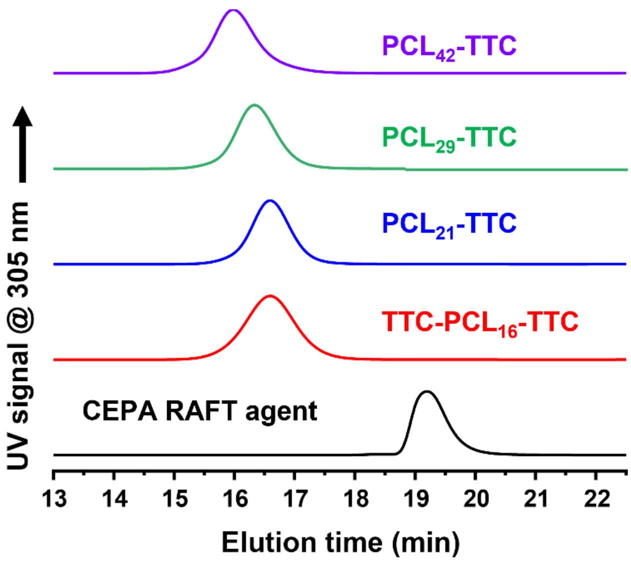

Figure S13. DMF GPC curves (UV detector set at 305 nm) recorded for TTC-PCL_{16}-TTC, PCL_{21}-TTC, PCL_{29}-TTC, PCL_{42}-TTC, and the CEPA RAFT agent. For the GPC analysis of CEPA, a DMF eluent containing 1% glacial acetic acid with no LiBr was used to ensure the carboxylic acid group of the RAFT agent was not ionized.

Supplementary Characterization Data for (PDMAC-)PCL-PDMAC Copolymers

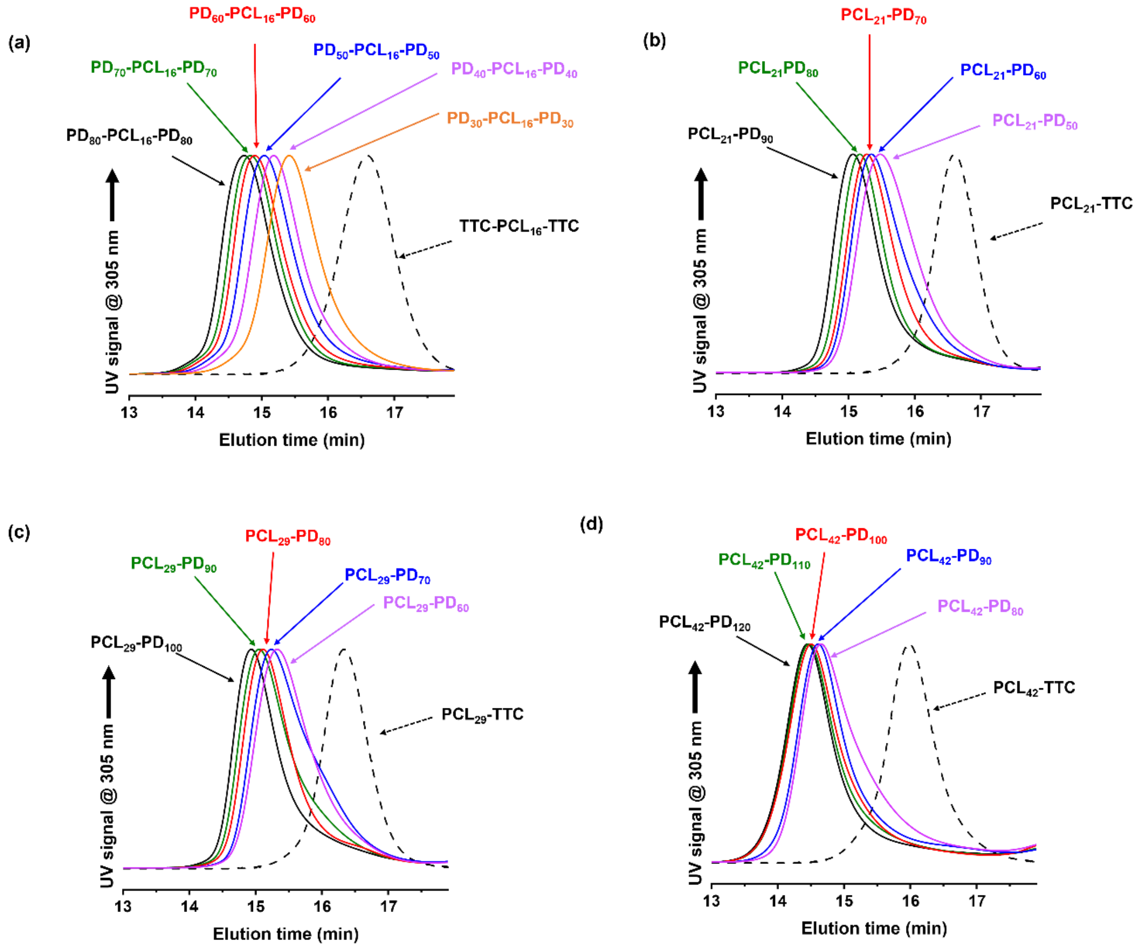

Figure S14. DMF GPC curves (UV detector at 305 nm) recorded for a series of block copolymers prepared by *reverse sequence* PISA in aqueous media using an ACVA initiator at 80 °C. (a) Bifunctional TTC-PCL_{16}-TTC precursor and a corresponding series of $PDMAC_x$-PCL_{16}-$PDMAC_x$ triblock copolymers. (b) Monofunctional PCL_{21}-TTC precursor and a corresponding series of PCL_{21}-$PDMAC_z$ diblock copolymers. (c) Monofunctional PCL_{29}-TTC precursor and a corresponding series of PCL_{29}-$PDMAC_z$ diblock copolymers. (d) Monofunctional PCL_{42}-TTC precursor and a corresponding series of PCL_{42}-$PDMAC_z$ diblock copolymers.

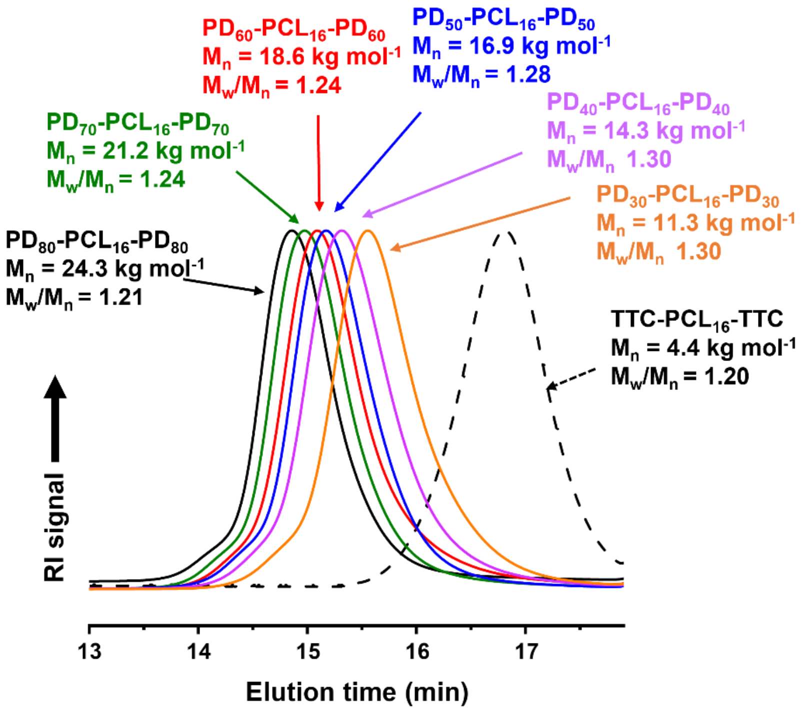

Figure S15. DMF GPC curves (refractive index detector) recorded for TTC-PCL_{16}-TTC and the corresponding series of $PDMAC_x$-PCL_{16}-$PDMAC_x$ triblock copolymers prepared by *reverse sequence* PISA (initially in 80% w/w aqueous solution prior to dilution to 10% w/w) using an ACVA initiator at 80 °C.

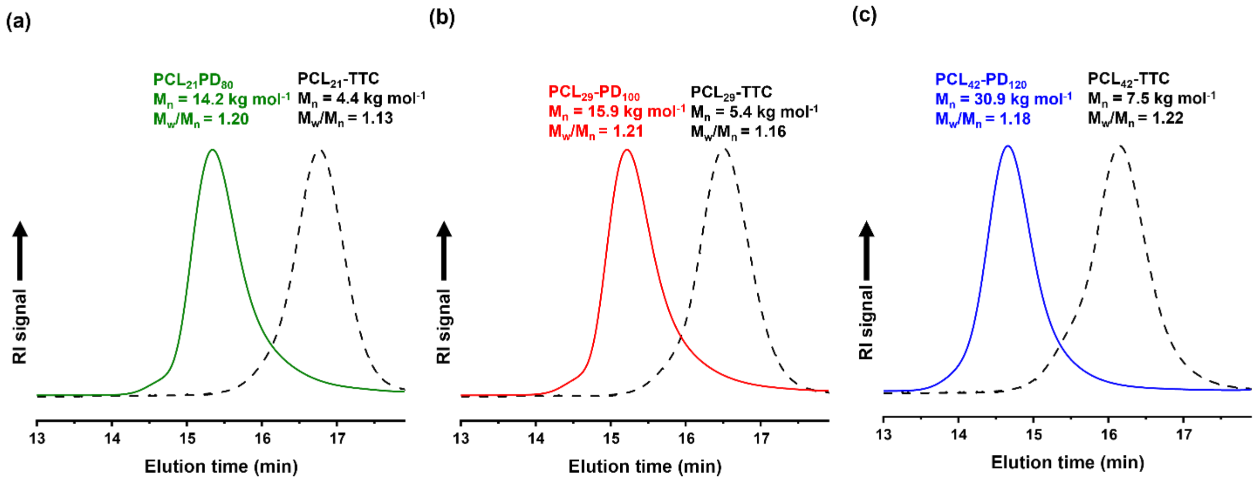

Figure S16. DMF GPC curves (refractive index detector) recorded for (a) PCL_{21}-TTC and a PCL_{21}-$PDMAC_{80}$ copolymer, (b) PCL_{29}-TTC and a PCL_{29}-$PDMAC_{100}$ copolymer and (c) PCL_{42}-TTC and a PCL_{42}-$PDMAC_{120}$ copolymer prepared by *reverse sequence* PISA (initially in 80% w/w aqueous solution prior to dilution to 10% w/w) using an ACVA initiator at 80 °C.

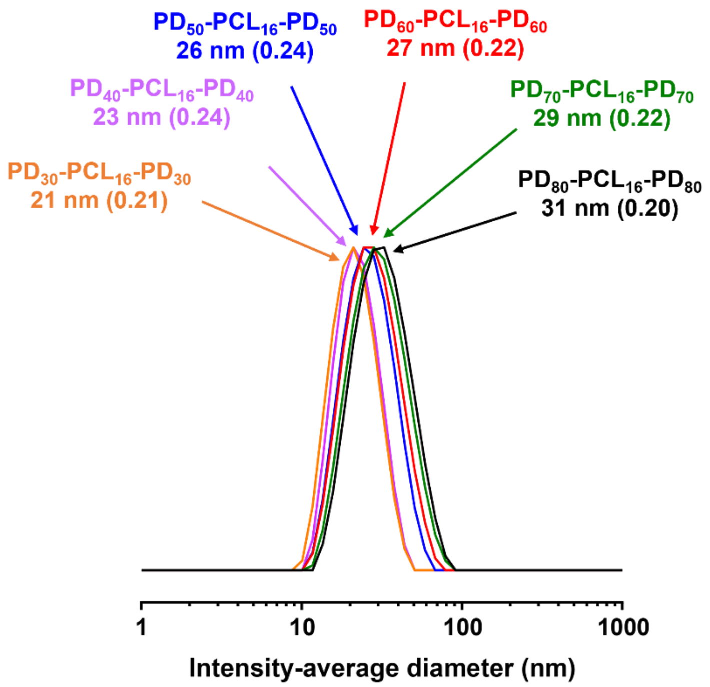

Figure S17. DLS particle size distributions recorded for a series of aqueous dispersions of $PDMAC_x$-PCL_{16}-$PDMAC_x$ nanoparticles (where x = 30, 40, 50, 60 or 80) prepared via *reverse sequence* PISA (initially conducted in the bulk, followed by dilution with deionized water to 10% w/w solids).

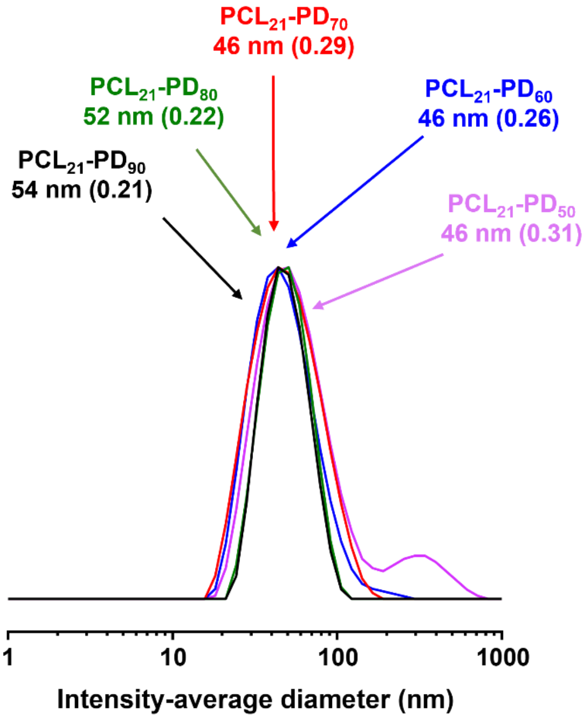

Figure S18. DLS particle size distributions recorded for a series of aqueous dispersions of PCL_{21}-$PDMAC_z$ nanoparticles (where x = 50, 60, 70, 80 or 90) prepared via *reverse sequence* PISA (initially conducted in the bulk, followed by dilution with deionized water to 10% w/w solids).

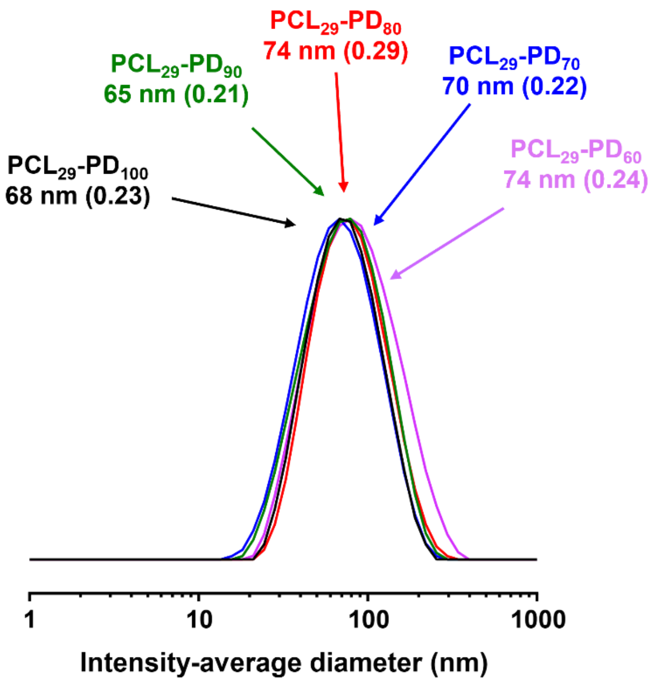

Figure S19. DLS particle size distributions recorded for a series of aqueous dispersions of PCL_{29}-$PDMAC_z$ nanoparticles (where x = 60, 70, 80, 90 or 100) prepared via *reverse sequence* PISA (initially conducted in the bulk, followed by dilution with deionized water to 10% w/w solids).

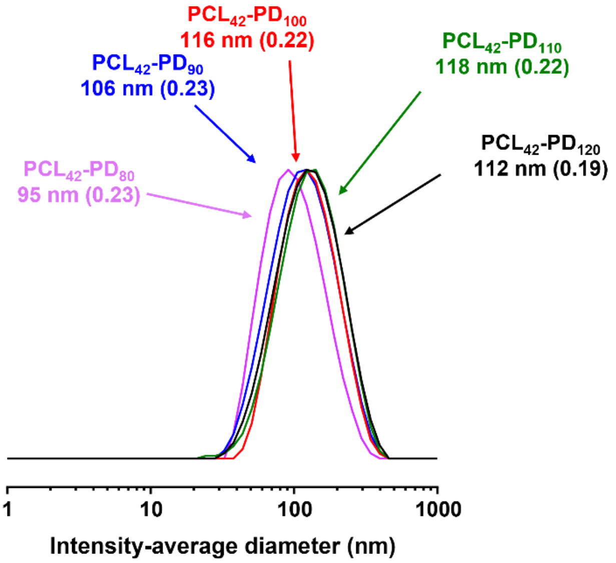

Figure S20. DLS particle size distributions recorded for a series of aqueous dispersions of PCL_{42}-$PDMAC_z$ nanoparticles (where x = 80, 90, 100, 110 or 120) prepared via *reverse sequence* PISA (initially conducted in the bulk, followed by dilution with deionized water to 10% w/w solids).

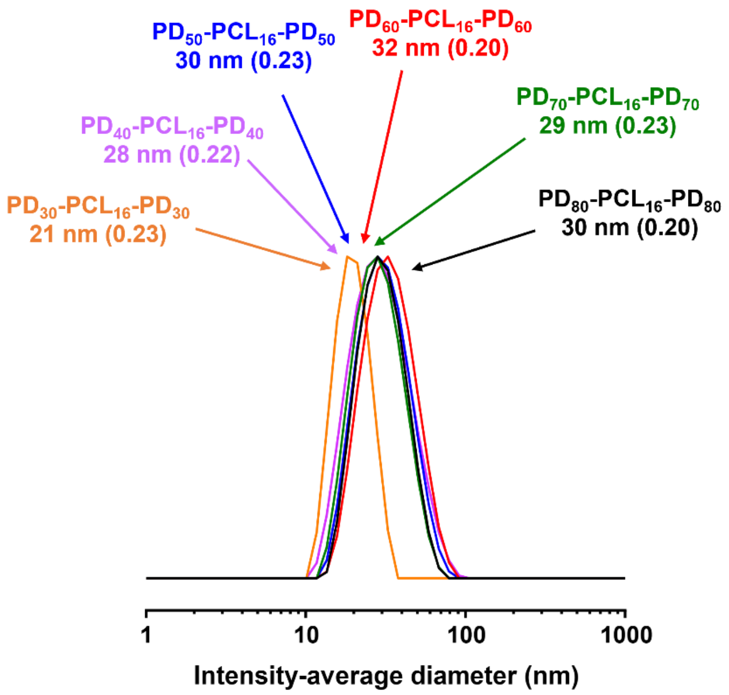

Figure S21. DLS particle size distributions recorded for a series of aqueous dispersions of PDMAC$_x$-PCL$_{16}$-PDMAC$_x$ nanoparticles (where x = 30, 40, 50, 60, 70 or 80) prepared via *reverse sequence* PISA (initially conducted at 80% w/w solids, followed by dilution with deionized water to 10% w/w solids).

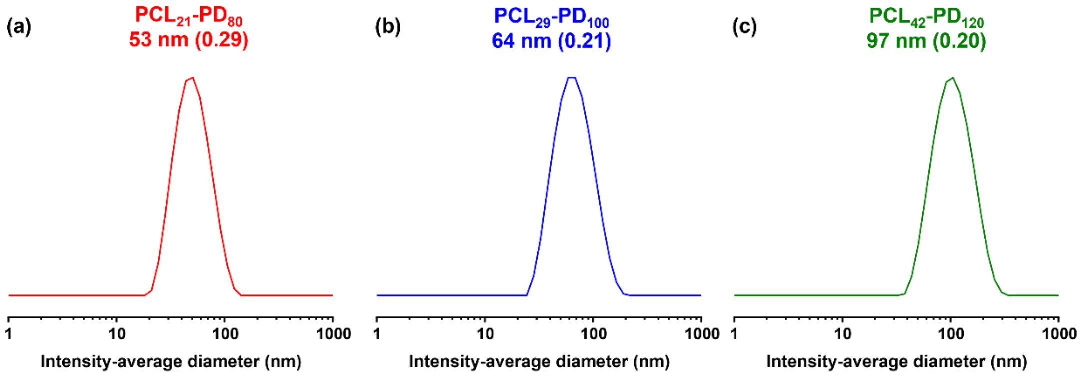

Figure S22. DLS particle size distributions recorded for a series of aqueous dispersions of (a) PCL$_{21}$-PDMAC$_{80}$, (b) PCL$_{29}$-PDMAC$_{100}$ and (c) PCL$_{42}$-PDMAC$_{120}$ nanoparticles via *reverse sequence* PISA (initially conducted at 80% w/w solids, followed by dilution with deionized water to 10% w/w solids).

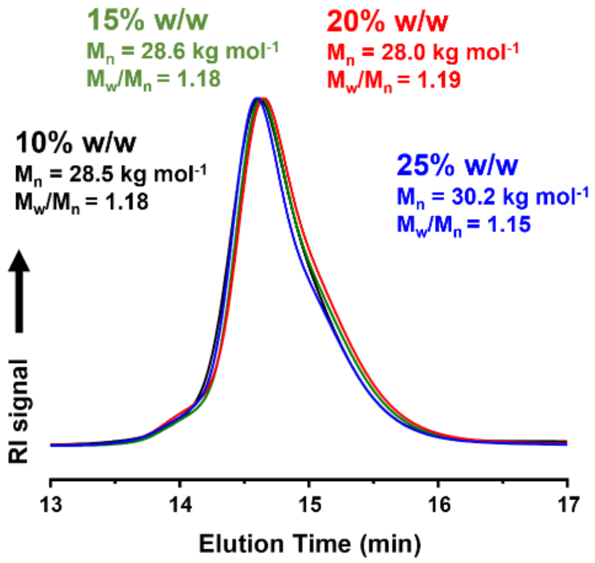

Figure S23. DMF GPC curves (refractive index detector) recorded for a series of $PDMAC_{80}$-PCL_{16}-$PDMAC_{80}$ triblock copolymers prepared by *reverse sequence* PISA (initially conducted in the bulk prior to dilution to 10-25% w/w solids) using an ACVA initiator at 80 °C.

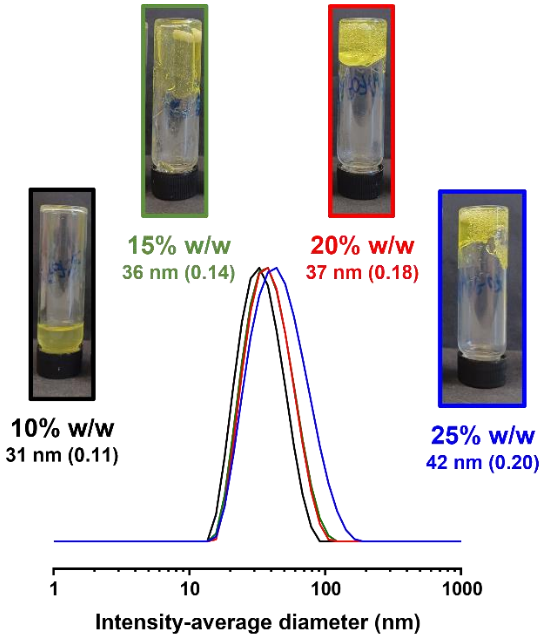

Figure S24. DLS particle size distributions recorded for $PDMAC_{80}$-PCL_{16}-$PDMAC_{80}$ nanoparticles prepared at a final concentration of 10% w/w (black data), 15% w/w (green data), 20% w/w (red data) and 25% w/w (blue data). Inset digital photographs show the physical appearance of each dispersion.

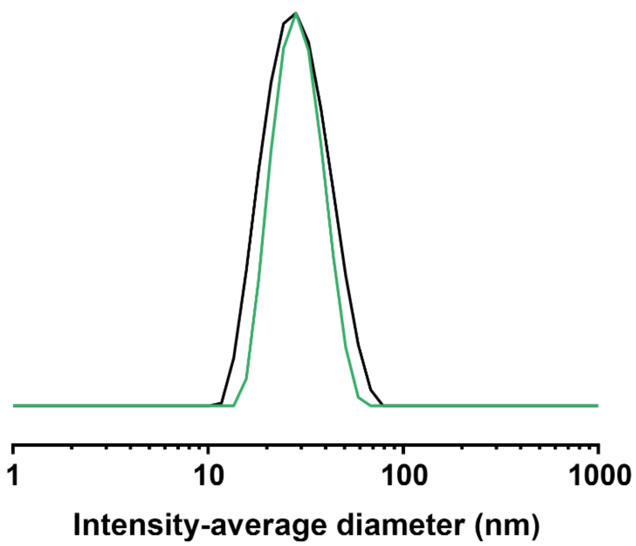

Figure S25. DLS particle size distributions recorded for a 0.1% w/w aqueous dispersion of $PDMAC_{50}$-PCL_{16}-$PDMAC_{50}$ nanoparticles immediately after synthesis (black curve) and after storage for 12 weeks as a 10% w/w dispersion (pH 6.7) at 20 °C (green curve).

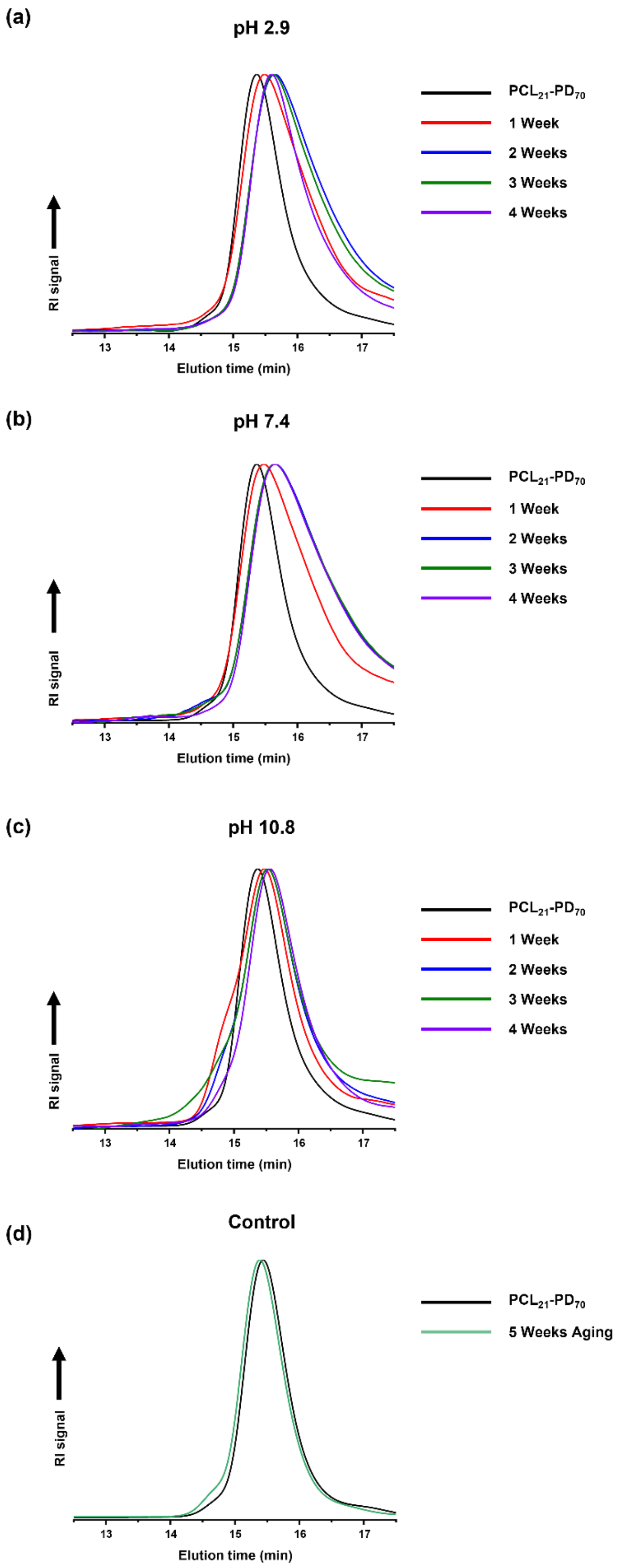

Figure S26. DMF GPC curves (refractive index detector) recorded during the hydrolytic degradation of a 1.0% w/w aqueous dispersion of PCL_{21}-$PDMAC_{70}$ nanoparticles at 37 °C for 1-4 weeks at (a) pH 2.9, (b) pH 7.4 (PBS buffer), (c) pH 10.8 and (d) DMF GPC curves (refractive index detector) recorded for a control experiment where of PCL_{21}-$PDMAC_{70}$ nanoparticles are stored in deionized water (10 % w/w, pH 6.7) at 20 °C for 5 weeks.

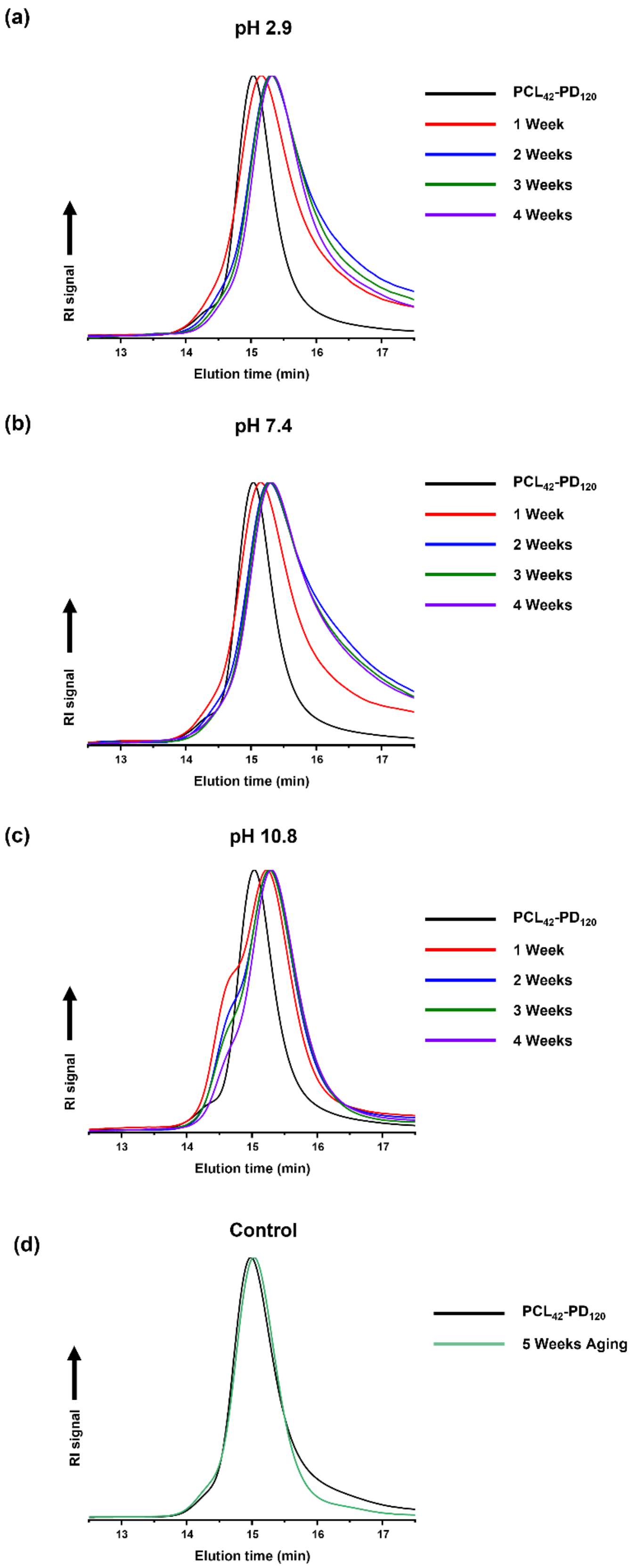

Figure S27. DMF GPC curves (refractive index detector) recorded during the hydrolytic degradation of a 1.0% w/w aqueous dispersion of PCL_{42}-$PDMAC_{120}$ nanoparticles at 37 °C for 1-4 weeks at (a) pH 2.9, (b) pH 7.4 (PBS buffer), (c) pH 10.8 and (d) DMF GPC curves (refractive index detector) recorded for a control experiment where of PCL_{42}-$PDMAC_{120}$ nanoparticles are stored in deionized water (10 % w/w, pH 6.7) at 20 ^{o}C for 5 weeks.

References

[54] M. Danial, S. Telwatte, D. Tyssen, S. Cosson, G. Tachedjian, G. Moad and A. Postma, *Polym. Chem.*, 2016, **7**, 7477–7487.

[55] B. G. G. Lohmeijer, R. C. Pratt, F. Leibfarth, J. W. Logan, D. A. Long, A. P. Dove, F. Nederberg, J. Choi, C. Wade, R. M. Waymouth and J. L. Hedrick, *Macromolecules*, 2006, **39**, 8574–8583.
